# Mechanistic Disruption of the TREM2–DAP12 Transmembrane Complex by Alzheimer’s Disease Mutations: A Multiscale Simulation Study

**DOI:** 10.1021/acs.jcim.5c01891

**Published:** 2025-11-26

**Authors:** Zhiwen Zhong, Martin Ulmschneider, Christian D. Lorenz

**Affiliations:** † Department of Chemistry, 4616 King’s College London, London SE1 1DB, U.K.; ‡ Department of Physics, King’s College London, London WC2R 2LS, U.K.; § Department of Engineering, 4616 King’s College London, London WC2R 2LS, U.K.

## Abstract

Triggering receptor expressed on myeloid cell 2 (TREM2) is an immunomodulatory receptor that plays a critical role in microglial activation through its association with the adaptor protein DNAX-activation protein 12 (DAP12). Variants in TREM2 have been implicated as genetic risk factors for Alzheimer’s disease (AD), most notably the extracellular domain variant R47H. However, recent studies highlight that transmembrane domain (TMD) mutations, including W191X in isoform-219 of TREM2, may also increase AD risk. Nonetheless, the molecular mechanisms underlying the TREM2–DAP12 complex formation and the role of specific TMD mutations in disease pathology remain unclear. Here, we employ multiscale molecular dynamics (MD) simulations to investigate the structural and dynamic effects of key TREM2 TMD mutations on the complex formed with DAP12 within a lipid bilayer composed of a POPC:cholesterol (80:20) mixture. Specifically, we analyzed four mutations in isoform-230 (K186A, K186X, W194A, W194X) and three constructs in isoform-219 (wild type, W191A, W191X). Our previous studies identified that K186 forms a critical salt bridge with DAP12 residue D50 in isoform-230. In this study, we extend this understanding by combining atomistic simulations with unsupervised machine learning approaches to analyze conformational changes across mutant complexes. Across variants, we observe isoform- and mutation-specific effects on helix orientation, hydrogen bonding, and electrostatic interactions that destabilize complex formation. This study provides atomistic-level insight into how disease mutations perturb membrane protein signaling interfaces and introduces a robust simulation and data-driven framework for studying transmembrane complexes involved in neurodegeneration and immunoreceptor function.

## Graphical abstract

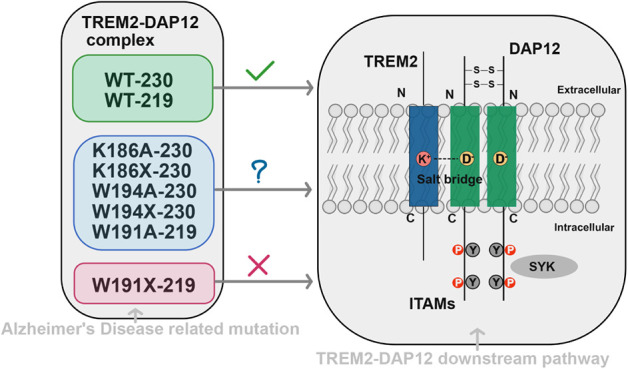

## Introduction

Alzheimer’s disease (AD) is a progressive neurodegenerative condition characterized mainly by β-amyloid (Aβ) plaque deposition in the brain.^1,2^ Microglia, the brain’s resident immune cells, are frequently found surrounding Aβ plaques in both AD patients^3^ and transgenic mouse models,^4^ where they contribute to the clearance of Aβ and the modulation of neuroinflammation. Recent genome-wide association studies (GWAS) have identified rare variants in TREM2 (Triggering Receptor Expressed on Myeloid cells 2) as significant genetic risk factors for late-onset AD, with the R47H mutation in the extracellular domain conferring a particularly strong effect.^1,5^ TREM2 functions as an immunomodulatory receptor on microglial surfaces and initiates intracellular signaling via its transmembrane adaptor DAP12 (DNAX-activating protein of 12 kDa).^6,7^

TREM2 interacts with a diverse range of ligands, such as phospholipids, sulfatides, bacterial lipopolysaccharide (LPS), and DNA,^8–11^ and the nature of these interactions can differentially modulate its signaling strength and outcome.^12^ Ligand engagement at the TREM2 extracellular domain initiates the signaling cascade through association with adaptor proteins DAP10/12.^13^ Subsequently, the TREM2 ectodomain is shed by ADAM10/17 metalloproteases, followed by intramembrane cleavage of the C-terminal fragment (CTF) by γ-secretase—a process whose structural mechanism has recently been elucidated by cryo-EM.^14–16^ This proteolytic sequence effectively terminates TREM2 signaling, enabling microglia to revert to a homeostatic state. Following ligand binding, TREM2 couples with DAP12 via their transmembrane domains,^17–19^ triggering phosphorylation of DAP12’s immunoreceptor tyrosine-based activation motifs (ITAMs) and recruitment of kinases, including SYK, PLCγ, and PI3K.^20–22^ Recent computational and structural studies have further highlighted how pathogenic TREM2 mutations modulate ligand recognition and small-molecule binding.^23,24^

The TREM2–DAP12 interaction is initiated through electrostatic pairing between lysine 186 (K186) of TREM2 and conserved aspartate residues (D50) in DAP12, forming a stable trimeric transmembrane complex.^25^ Additional stabilization is provided by W194 in TREM2, which forms a hydrogen bond with T54 in DAP12.^26^ While most functional studies of TREM2 focus on extracellular domain mutations, emerging evidence implicates transmembrane domain variants in AD risk. Notably, the W191X (X represents truncation) nonsense mutation in TREM2 isoform-219 has been associated with increased AD susceptibility in African American populations.^27^ TREM2 exists in at least three isoforms by alternative splicing^28–30^ with isoform 1 (230 residues) being the canonical and most abundant form.^31^ Isoform 2 (219 residues) was found to be expressed at lower levels than isoform 1 in the hippocampus of AD patients.^32^ Isoform 3 (222 residues) remains poorly characterized at the protein level and lacks structural data;^33^ hence, our analysis focused on isoforms 1 and 2 for which validated sequence and topology information are available.

Structural biology approaches like cryo-EM and X-ray crystallography offer valuable but static views of protein complexes.^34–36^ Machine-learning-based prediction models, such as AlphaFold2 and AlphaFold3,^37,38^ provide high-quality structures but often lack the conformational dynamics critical for understanding membrane-embedded interactions.^39^ Atomistic molecular dynamics (MD) simulations offer complementary insights by capturing time-resolved structural fluctuations within biologically realistic environments.^40,41^ In this study, we build upon our previous work resolving the TREM2–DAP12 complex in a lipid bilayer,^26^ investigating the structural consequences of AD-associated and designed point mutations in the TREM2 transmembrane domain (TMD). We examined six variants: K186A and K186X (isoform 230), W194A and K194X (isoform 230), and W191A and W191X (isoform 219), each positioned at the DAP12-binding interface and linked to disrupted binding affinity with DAP12. K186 (isoform 230) forms the canonical salt bridge with DAP12 D50, W194 stabilizes the hydrophobic interface through aromatic contacts, and W191 (isoform 219) corresponds to this motif and is truncated in the pathogenic W191Ter mutation. These residues thus capture both structural and genetic determinants of DAP12 binding. All simulations are conducted with the TREM2–DAP12 complex embedded within a model lipid membrane consisting of an 80:20 mixture of POPC and cholesterol. By combining multiscale MD simulations with unsupervised machine learning analysis, we provide an atomistic perspective on how these mutations modulate the TREM2–DAP12 complex stability and their potential implications in Alzheimer’s disease pathogenesis.

## Results

### Unsupervised Learning Reveals Distinct Conformational Clusters in TREM2–DAP12 Complexes

Previous studies have shown that TREM2 interacts with the signaling adaptor DAP12, primarily through a key salt bridge between K186 in TREM2 and D50 in DAP12.^7,17^ The cartoons shown in Figure 1A represent the membrane-embedded α-helices structures of TREM2 and DAP12, which we reported in previous work.^26^ A focused sequence alignment of the transmembrane regions is presented in Figure 1B (with the full alignment shown in Supplemental Figure S1). We observed that residue W191 in the TREM2 isoform-219 corresponds positionally to residue K186 in TREM2 isoform-230. Since K186 is known to be critical for TREM2–DAP12 interaction and W191 has been potentially linked to Alzheimer’s disease, this suggests that W191 may play a key role in mediating the interaction between TREM2 isoform-219 and DAP12.

**Figure 1:**
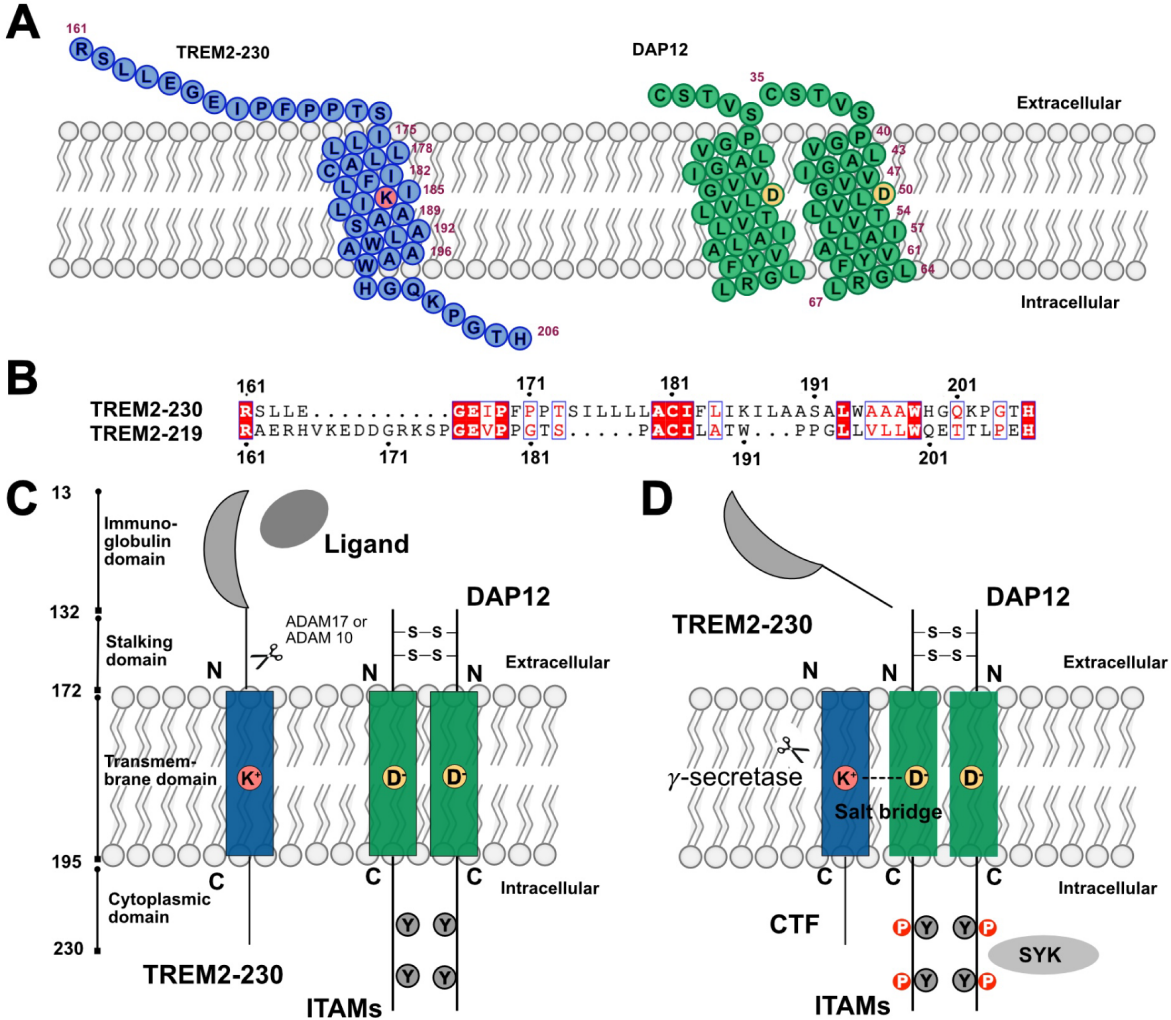
TREM2–DAP12 complex: sequence features and signaling mechanisms. (A) Schematic of the transmembrane domains of TREM2 (isoform 1, 230 aa) and the DAP12 dimer, highlighting key residues involved in membrane embedding and interaction. (B) Sequence alignment of TREM2 isoform 1 (230 aa) and isoform 2 (219 aa), emphasizing differences in the transmembrane regions. (C) Ligand engagement induces conformational changes in TREM2, followed by proteolytic cleavage of the ectodomain by ADAM10/17. (D) TREM2–DAP12 association via salt bridges between transmembrane residues triggers downstream phosphorylation of DAP12 ITAM motifs, leading to recruitment of SYK and other kinases.

Figure 1C illustrates the ligand binding to the extracellular domain of TREM2 and the subsequent intracellular signaling cascade initiated by DAP12. Upon proteolytic cleavage and formation of the C-terminal fragment (CTF), TREM2 remains associated with DAP12, enabling ITAM phosphorylation and SYK recruitment (Figure 1D). To investigate how specific mutations affect the structural dynamics of the TREM2–DAP12 complex, we introduced several point mutations into TREM2 and simulated each variant in complex with DAP12. The details of system preparation and mutation design are described in the Methods section. Coarse-grained molecular dynamics (CG-MD) simulations were performed for 300 μs on the TREM2 isoform-230 variants and 200 μs on the TREM2 isoform-219 variants.

Using the trajectories from the CG-MD simulations, we implemented an unsupervised machine learning pipeline to characterize the conformational landscapes of the TREM2–DAP12 complexes. High-dimensional contact map data from the trajectories were first embedded into a 2D space using the Uniform Manifold Approximation and Projection (UMAP) algorithm. Then, the Hierarchical-Density-Based Spatial Clustering of Applications with Noise (HDBSCAN) algorithm was applied to identify distinct conformational states in the UMAP embeddings. The resulting clusters represent the dominant structural states sampled by each mutant. These clusters are visualized in Figure 2A and D for the TREM2 isoform-230 and TREM2 isoform-219 constructs, respectively. The relative populations of these conformational states are depicted as doughnut plots in Figure 2B and E. The temporal evolution of the cluster assignments across the various simulation trajectories, shown in Figure 2C and F, reveals differences in the stability and dynamics of the sampled conformations of the various mutants. These results indicate different conformations on different time scales. The detailed contact maps and representative conformational structures are shown in Supplemental Figures S2–S8. These representations connect the unsupervised clustering results to physically meaningful structural observables.

**Figure 2:**
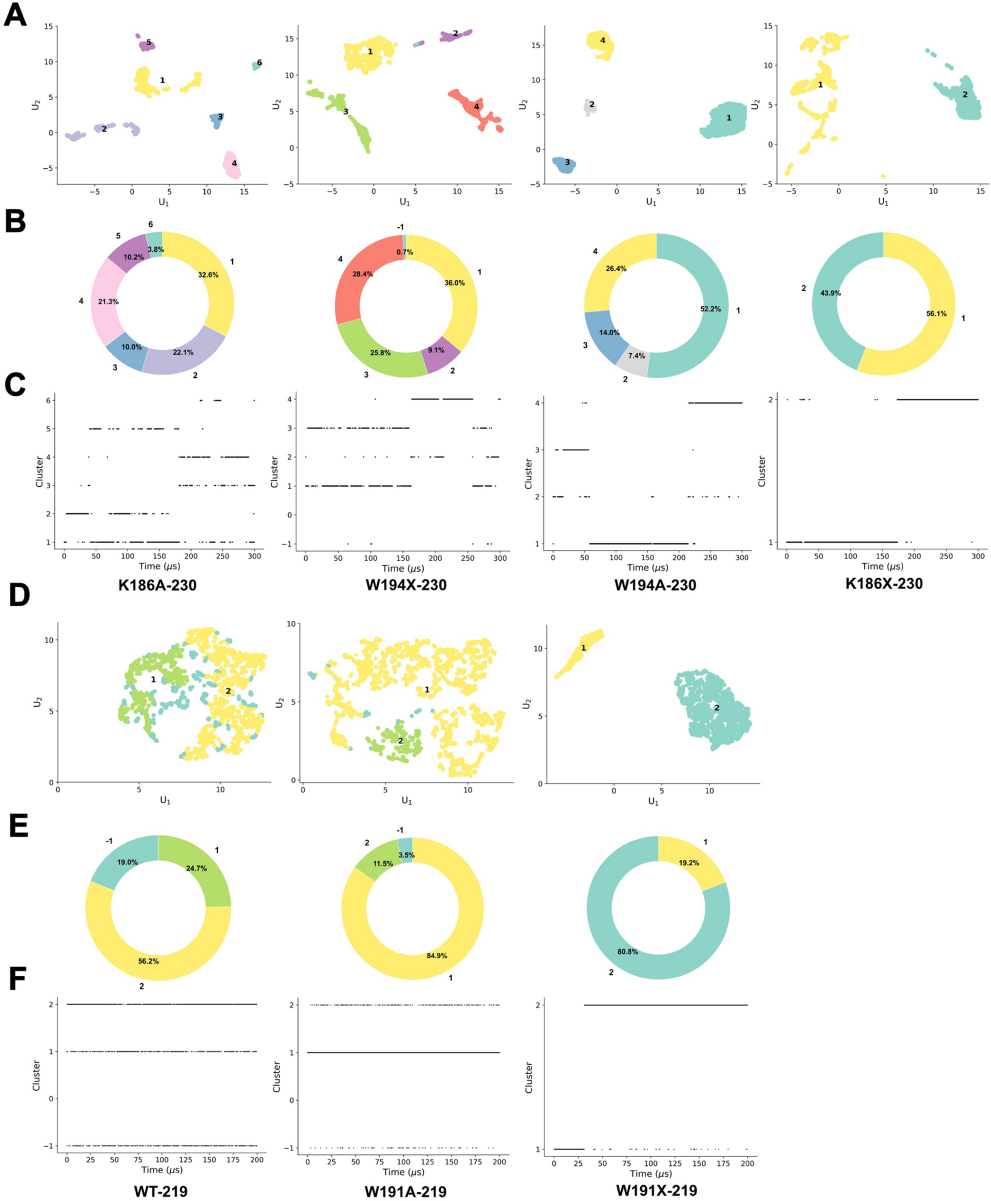
Unsupervised clustering reveals distinct conformational states of TREM2–DAP12 mutants. (A) UMAP (Uniform Manifold Approximation and Projection) plots showing the clustering of conformational states for four TREM2 isoform-230 mutants: K186A, W194X, W194A, and K186X. Each point represents a structural snapshot colored by its assigned cluster. (B) Corresponding doughnut charts depicting the relative abundance (percentage) of each conformational cluster identified in panel A. (C) Cluster assignments over time for each mutant in panel A, illustrating the temporal evolution and stability of conformational states across simulation trajectories. (D) UMAP plots of three TREM2 isoform-219 constructs: wild-type (WT), W191A, and W191X, showing the distribution of their structural ensembles. (E) Doughnut charts representing the relative population of clusters for the constructs shown in panel D, indicating how mutations alter conformational preferences. (F) Temporal evolution of cluster assignments for the TREM2 isoform-219 constructs shown in panel D, highlighting the stability and transitions between conformational states over time.

### Characterization of TREM2–DAP12 Interaction Interfaces and Mutant Effects

To further understand the interaction interfaces of the different TREM2–DAP12 complexes, we identified representative structures from the dominant conformational clusters observed in the CG-MD simulations (Table 1) and then converted them to all-atom models using the CHARMM-GUI converter.^42,43^ We simulated the resulting systems for 300 ns. From these trajectories, we constructed contact maps using a 3 Å cutoff distance^26^ between C-α atoms to define residue–residue interactions (Supplemental Figures S9–S15). Residue pairs that maintained contact for over 60% of the trajectory were labeled as interacting residues, while those with contact persistence above 90% were defined as important residues (Figure 3, blue and red dots, respectively). A comprehensive summary of the interacting residue pairs that persist for 60%, 70%, 80%, and 90% of the trajectories is provided in Supplemental Table S1.

**Figure 3:**
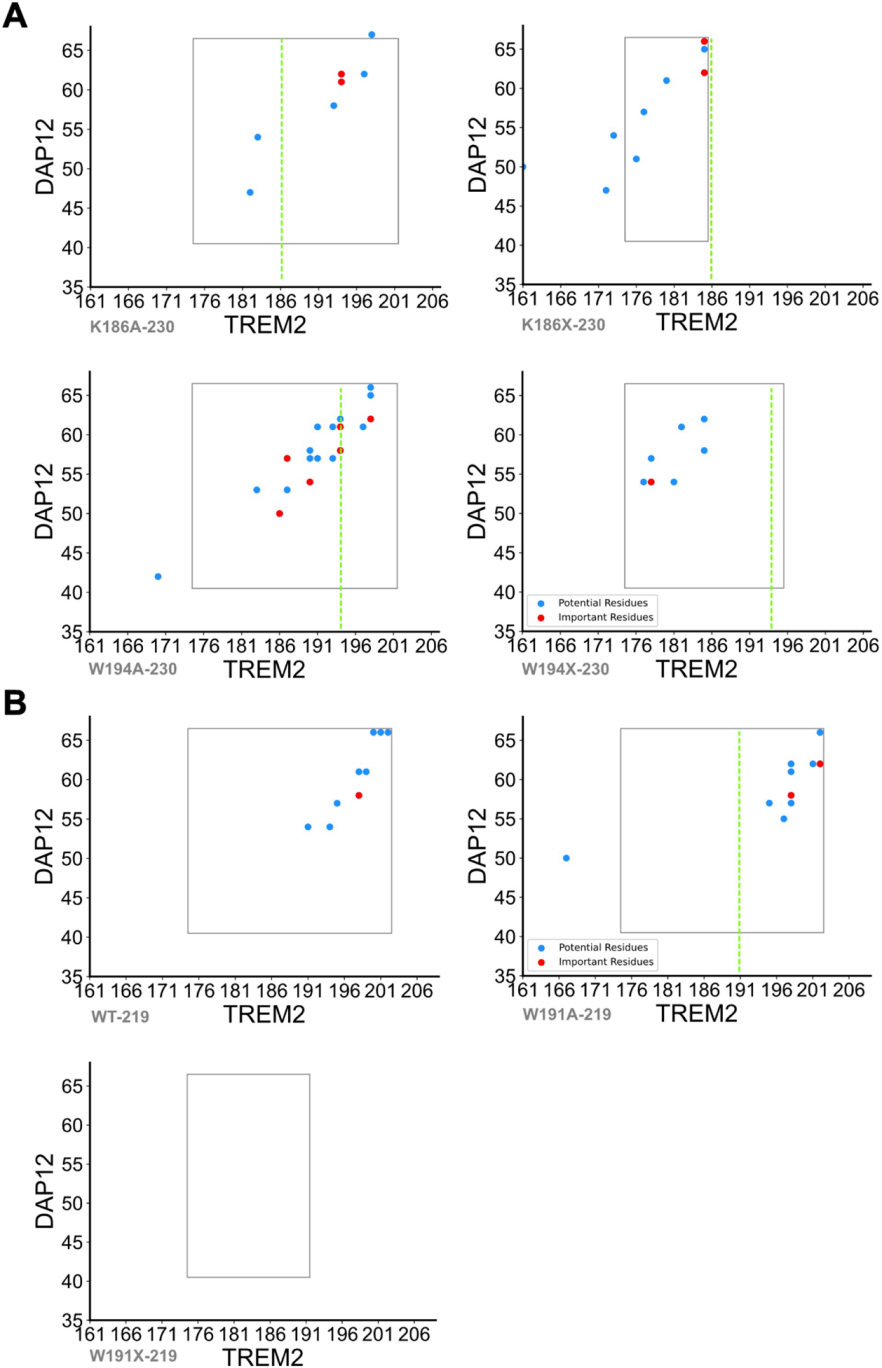
Residue-level interaction analysis of TREM2–DAP12 transmembrane mutants. All quantitative analyses were averaged over three independent replicas, which showed consistent trends in interface disruption and helix separation. Overlay versions of these contact maps, showing mutant-specific differences in contact frequency, are provided in Supplementary Figure S31. (A) Pairwise residue interaction maps for TREM2 mutants (K186A, K186X, W191A, W191X) in the 230 construct, plotted with TREM2 residues on the *x*-axis and DAP12 residues on the *y*-axis. Blue dots indicate interacting residues, and red dots highlight residues identified as important for the interaction. The green dashed line shows the mutation sites. (B) Residue interaction maps for selected TREM2 constructs in the 219 background: wild-type (WT), W191A, and W191X. These maps reveal the altered interaction profiles resulting from specific point mutations and truncations, underscoring the differential contribution of individual residues to the TREM2–DAP12 interface.

**Table 1:** Cluster Assignments and Times for Each Variant

Variant	Replica 1	Replica 2	Replica 3
K186A-230	Cluster 4	Cluster 1	Cluster 3
	290 μs	295 μs	298 μs
K186X-230	Cluster 2	Cluster 4	Cluster 4
	297 μs	299 μs	300 μs
W194A-230	Cluster 4	Cluster 4	Cluster 4
	297 μs	299 μs	300 μs
W194X-230	Cluster 2	Cluster 2	Cluster 2
	297 μs	299 μs	300 μs
WT-219	Cluster 1	Cluster 2	Cluster 2
	198 μs	199 μs	200 μs
W191A-219	Cluster 2	Cluster 1	Cluster 1
	198 μs	199 μs	200 μs
W191X-219	Cluster 2	Cluster 2	Cluster 2
	197 μs	198 μs	200 μs

In TREM2 isoform-230 (Figure 3A), the K186A mutant exhibited fewer persistent contacts than are found in the wild type, underscoring the role of K186 in stabilizing the TREM2–DAP12 complex. The key contact sites between the K186X mutant and DAP12 are found to be in the C-terminal regions of both proteins. Notably, the W194A-230 mutant displayed the highest number of contacts among the isoform 1 variants, with interaction hotspots spanning from the middle to the C-terminal region of TREM2 and across the whole length of DAP12. In contrast, the W194X-230 mutant showed markedly reduced contact frequency, suggesting impaired complex formation.

In TREM2 isoform-219 (Figure 3B), WT-219 complexes retained substantial interactions ranging from the middle- to the C-terminal regions of both TREM2 and DAP12. The W191A mutation preserved these contacts with a prominent clustering toward the C-terminal regions. Strikingly, the W191X-219 mutant exhibited no detectable contacts within the defined threshold, indicating a complete loss of interaction. This observation is consistent with the hypothesis that W191 is critical for maintaining the TREM2–DAP12 interface and is in line with genetic studies linking W191 variants to Alzheimer’s disease susceptibility.

The rectangles overlaid in Figure 3 denote the approximate boundaries of the transmembrane α-helices in each complex, providing structural context for the observed contact distributions.

### Mechanistic Insights into Residue–Residue Interactions and Mutant-Induced Disruptions in TREM2–DAP12 Complex Stability

To gain mechanistic insight into the specific residue–residue interactions stabilizing TREM2–DAP12 complexes, we analyzed interaction types across all mutant systems (Figure 4A). Within the K186A-230 mutant, we found that π–π stacking interaction between W194 of TREM2 and Y62 of DAP12 was observed in the K186A-230 mutant (Figure 4B) to play an important role in the formation of the complex, occurring 37.60% of the total simulation time-32.28% in a parallel (sandwich-like) orientation and 5.32% in a T-shaped configuration (Figure 4C). The details of the π–π stacking measurement can be found in Supplemental Figure S16. In addition, a hydrophobic interaction between W194 and V61 further supports the trimer stability (Figure 5A).

**Figure 4:**
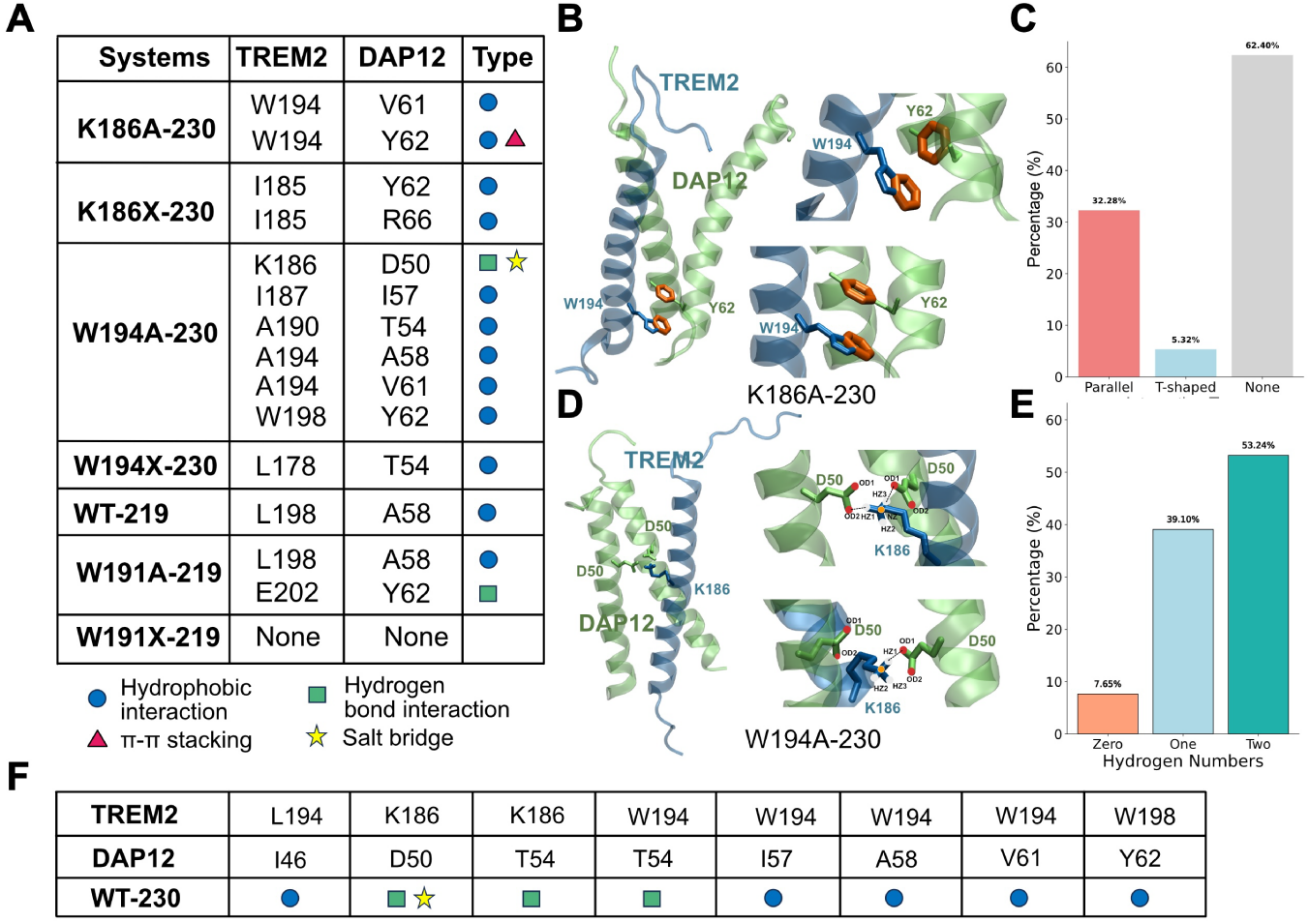
Molecular interaction profiles of TREM2–DAP12 transmembrane mutants. (A) Table summarizing key residue–residue interactions between TREM2 and DAP12 for various mutants and constructs. Interaction types are color-coded: hydrophobic (blue circle), π–π stacking (red triangle), hydrogen bonds (green square), and salt bridges (yellow star). (B) Representative structural models illustrating π–π stacking interactions between W194 (TREM2) and Y62 (DAP12) observed in K186A-230. (C) Bar graph quantifying the frequency of different π–π interaction geometries (parallel, T-shaped, or none) across simulation frames. (D) Structural snapshots showing the formation of a salt bridge between K186 (TREM2) and D50 (DAP12) in W194A-230. (E) Histogram showing the distribution of hydrogen bond numbers formed between K186 and D50 across simulations. These analyses demonstrate that specific point mutations significantly alter interaction patterns at the TREM2–DAP12 interface. (F) The table shows the important residue pairs of WT-230.

**Figure 5:**
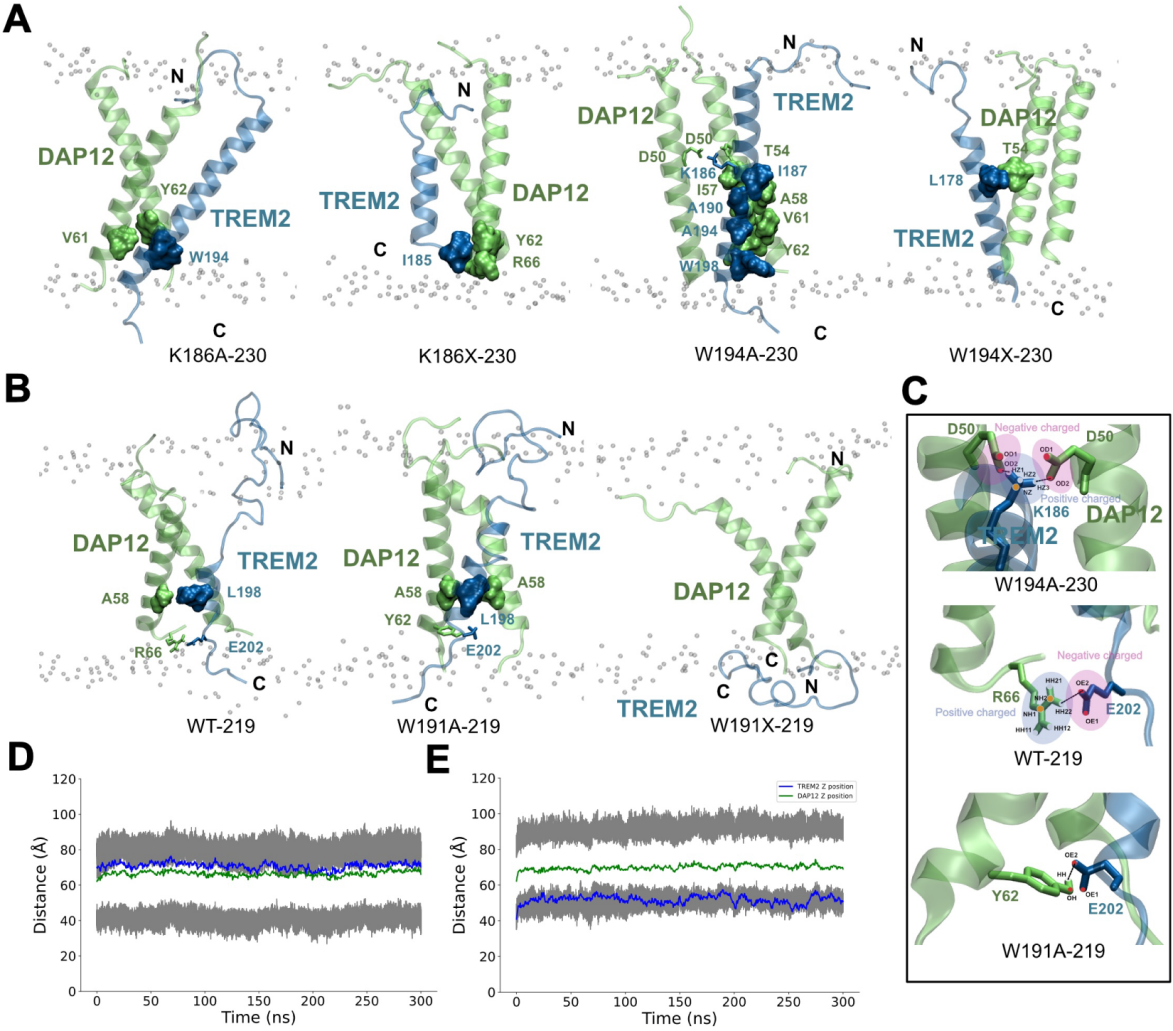
Structural insights into the TREM2–DAP12 transmembrane interactions and stability across mutants. (A) Representative snapshots of the TREM2–DAP12 transmembrane complexes embedded in a lipid bilayer for the TREM2 isoform-230 mutants: K186A, K186X, W194A, and W194X. Interacting residues at the interface are shown in surface representation. (B) Structural models of TREM2 isoform-219 constructs (WT, W191A, W191X) in complex with DAP12, highlighting the critical interfacial residues. (C) Zoomed-in views of key electrostatic and hydrogen-bond interactions formed between TREM2 and DAP12 residues in W194A-230, WT-219, and W191A-219. Positively and negatively charged regions are indicated. (D, E) Distance measurements over time between the center of mass of selected TREM2 and DAP12 transmembrane residues from molecular dynamics simulations. (D) The distance with the WT-219; (E) constructs with the W191X-219 construct. These structural and dynamic analyses reveal that specific point mutations and truncations significantly alter the interfacial interactions and complex stability.

In the W194A-230 mutant, a stable hydrogen bond forms between K186 in TREM2 and D50 in both DAP12 monomers (Figure 4D). This hydrogen bond persists for 92.34% of the simulation, with a hydrogen bond to one DAP12 monomer present 39.10% of the time and hydrogen bonds to both DAP12 monomers occurring simultaneously in 53.24% of the frames (Figure 4E). The details of the number of hydrogen bonds as a function of time can be found in Supplemental Figure S17. The further interaction network that stabilizes the W194A-230 complex includes multiple hydrophobic contacts and a salt bridge that contribute to tight trimer packing (Figure 5C, top panel). To facilitate comparison between the wild-type and mutant forms of isoform 1, the previously reported key interacting residues in WT-230 ^26^ are also highlighted in Figure 4F.

In contrast, the two truncated TREM2 isoform-230 complexes are primarily stabilized by hydrophobic interactions. In the case of the W194X-230 complex, the hydrophobic contact between L178 (TREM2) and T54 (DAP12) is found to play an important role in the stabilization of the complex. While in the K186X-230 complex, we found that the complex is a relatively stable conformation driven by hydrophobic interactions involving I185 of TREM2 with Y62 and R66 of DAP12 (Figure 5A).

For the WT-219 system, complex stability is maintained through a hydrophobic interaction between L198 (TREM2) and A58 (DAP12), along with a salt bridge and hydrogen bond between E202 (TREM2) and R66 (DAP12), with a 70% occupancy reported in Table S1 and shown in Figure 5C (middle panel). The W191A-219 mutant retains the L198-A58 hydrophobic interaction and features an additional hydrogen bond between E202 (TREM2) and Y62 (DAP12) (Figure 5C, bottom panel).

In stark contrast, the W191X-219 mutant completely disrupts all residue-level contacts, decreasing the complex stability (Figure 4A). Structural analysis reveals that TREM2 reorients horizontally along the membrane interface, while DAP12 retains a vertical orientation (Figure 5B). Analysis of the *z*-axis positioning over time (Figure 5D,E) further confirms this dissociation: in WT-219, both proteins remain colocalized in the upper membrane leaflet, whereas in W191X-219, TREM2 shifts toward the lower leaflet, while DAP12 remains within the center of the membrane.

The K186X-230 and W191X-219 mutants exhibited significantly greater center-of-mass distances between TREM2 and DAP12 in the *x* –*y* plane, measuring 13.02 and 11.20 Å, respectively (Supplementary Figure S18). In contrast, smaller distances were observed in the K186A-230 (7.86 Å), W194A-230 (8.08 Å), W194X-230 (9.83 Å), WT-219 (6.06 Å), and W191A-230 (6.07 Å) complexes. These findings suggest that the increased spatial separation in K186X-230 and W191X-230 reflects destabilized or weakened interactions between TREM2 and DAP12.

### Impact of Point Mutations on Structural Flexibility and Stability of the TREM2–DAP12 Complex

To investigate how point mutations affect structural flexibility, we analyzed the root-mean-square fluctuation (RMSF) profiles of each TREM2–DAP12 complex (Figure 6). Figure 6A shows the RMSF values for the 230-residue constructs. Traces are replica-averaged after least-squares alignment to the DAP12 transmembrane Cα atoms; shaded envelopes indicate standard deviations, and colored symbols mark the mutation sites in the same colors as the mutant traces. Compared to the WT-230 complex, the K186A-230 mutant displays increased flexibility in the N-terminal region of TREM2. This likely results from the loss of K186-mediated anchoring interactions. In the K186X-230 complex, higher fluctuations are observed in one of the DAP12 chains, suggesting that truncation of TREM2 disrupts the symmetric stability of the trimeric interface. The W194A-230 variant also exhibits elevated dynamics in the N-terminal domain of TREM2, possibly due to the loss of the aromatic W194 residue, which contributes to π-stacking and hydrophobic stabilization. W194X-230 also shows localized fluctuation increases, especially toward the C-terminal region, indicating reduced stability.

**Figure 6:**
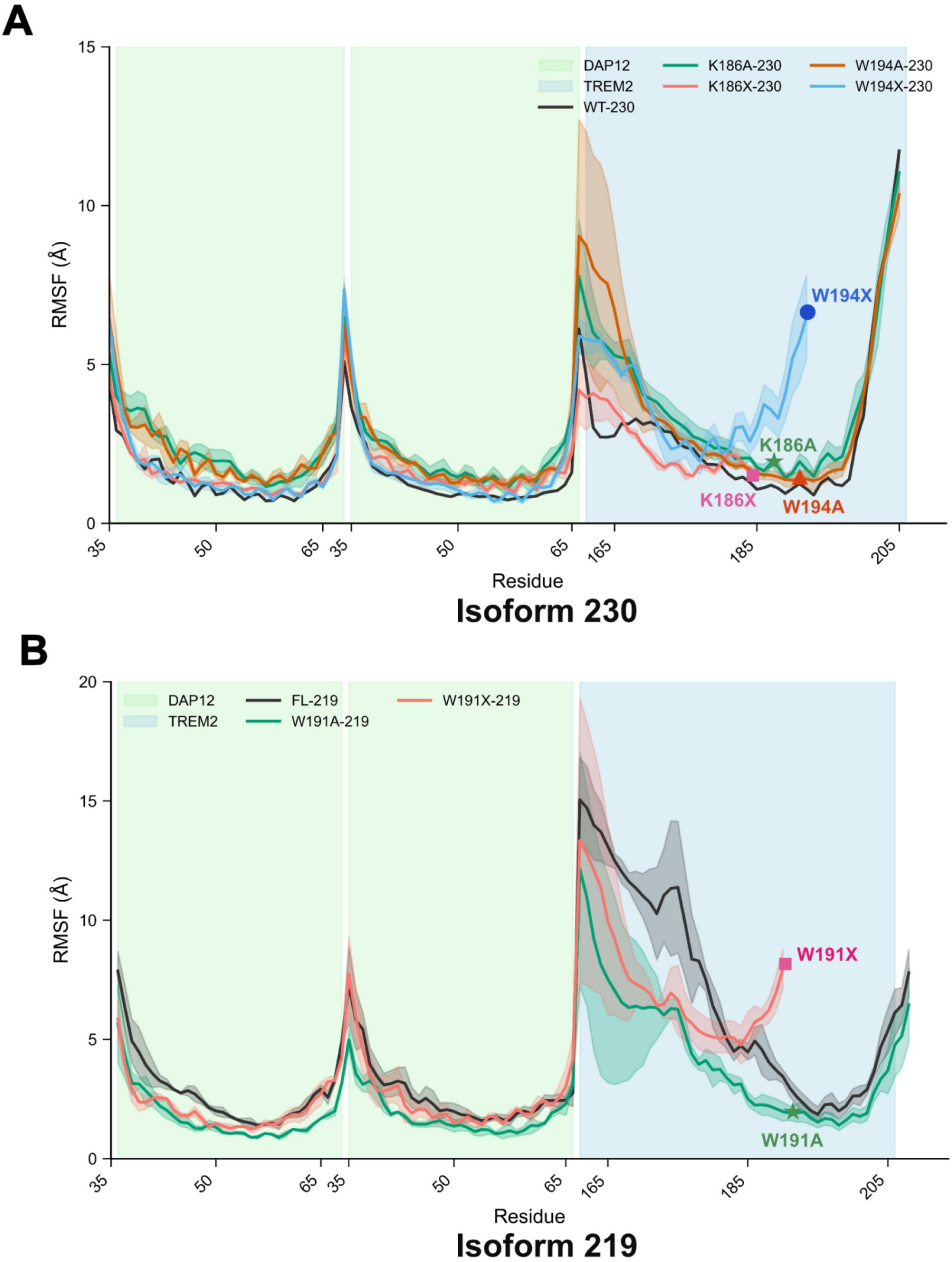
Root mean square fluctuation (RMSF) profiles of TREM2–DAP12 complexes show mutation-dependent flexibility changes and annotated mutation sites. (A) RMSF traces of the isoform-230 complexes comparing WT-230 and mutants (K186A, K186X, W194A, W194X). Curves represent the mean RMSF across three replicas after least-squares alignment to the DAP12 transmembrane Cα atoms; shaded areas indicate standard deviations. Green and blue background regions mark DAP12 and TREM2 transmembrane segments, respectively. Mutation sites are highlighted by colored symbols (stars, rectangles, circles, and triangles) matching each mutant trace, indicating the residue positions where the mutations occur. (B) RMSF profiles for isoform-219 complexes, including WT-219, W191A-219, and W191X-219. The shaded envelopes show replica variability, and colored symbols mark the corresponding mutation sites using the same color scheme as panel A. Together, these results demonstrate that C-terminal truncations (K186X, W191X) increase local flexibility, consistent with complex destabilization.

In the 219-residue systems (Figure 6B), replica-averaged RMSF profiles with shaded variability are shown, and mutation sites are indicated by colored symbols consistent with panel A. The WT-219 complex exhibits extensive flexibility in the cytoplasmic region of TREM2 and the DAP12 interface, maybe due to the lack of interaction of the N-terminus of TREM2 with either DAP12 or lipids (Figure 5B). W191A-219 displays a slightly more rigid profile, possibly due to compensatory stabilizing hydrogen bonds. W191X-219 shows moderate flexibility across both proteins at the N-terminus, with more pronounced fluctuations toward the C-terminus. These RMSF profiles underscore the dynamic nature of the TREM2–DAP12 interaction and reveal how specific mutations perturb stability through altered flexibility in both transmembrane and cytoplasmic domains.

## Discussion

The W191X mutation introduces a premature stop codon within the transmembrane domain of the TREM2 isoform-219, leading to truncation of the receptor’s C-terminal tail. Genetic studies have previously associated this nonsense variant with an increased risk of Alzheimer’s disease, particularly in African American populations.^27,30^ However, the biophysical consequences of W191X on TREM2–DAP12 complex formation and function have remained elusive.

Our simulations provide structural and mechanistic insights into how this mutation disrupts interprotein signaling. In contrast to the WT-219 and W191A-219 systems, which maintain robust interactions through hydrophobic, hydrogen bond, and salt bridge networks, the W191X-219 complex shows a complete loss of residue-level contacts at the TREM2–DAP12 interface (Figure 3B and Figure 4A). This disruption is further supported by the W191X variant, which exhibits a striking decoupling of the two helices, both laterally (11.2 Å in the *x*–*y* plane) and vertically (along the *z*-axis) orientation (Figure 5D,E and Supplemental Figure S18). Notably, the TREM2 helix in W191X-219 reorients almost horizontally along the bilayer interface—a configuration incompatible with canonical receptor-adaptor signaling.^6,7^

At the molecular level, the truncation eliminates distal cytoplasmic residues, including E202—a key site for salt bridge formation with R66 of DAP12 in WT-219 and W191A-219 systems (Figure 5C). The loss of this interaction likely destabilizes the helix alignment necessary for signal transduction via ITAMs on DAP12. These findings collectively suggest that W191X disrupts not only static contacts but also the dynamic coherence required for functional signaling. Our simulations targeted the transmembrane signaling module, corresponding to the C-terminal fragment that remains after ectodomain shedding, which is a biologically relevant form for DAP12 interaction. Inclusion of the extracellular domain could influence helix tilt or dynamics, but this is not discussed here and could be explored in future work.

Importantly, our results also provide comparative insights into mutation-induced destabilization across isoforms. The K186X-230 mutant, though distinct in sequence and location, mirrors the disruption seen in W191X-219, with increased center-of-mass separation and reduced interhelical stability (Figure 5A and Supplemental Figure S18). This parallel underscores the critical role of C-terminal residues in preserving the spatial geometry of the receptor-adaptor complex.

Beyond the immediate biophysical implications, these findings have broader relevance for understanding TREM2’s role in neuroimmune signaling. Misalignment or dissociation of TREM2 from DAP12 could impair SYK recruitment and ITAM phosphorylation, mechanisms essential for microglial activation and phagocytosis.^44,45^ Our simulations focus on the preshedding, signaling-competent TREM2–DAP12 trimer that mediates DAP12 ITAM phosphorylation. Following ectodomain shedding by ADAM proteases, the resulting monomeric TREM2 C-terminal fragment (CTF) becomes the substrate for γ-secretase cleavage. These stages are mechanistically distinct: γ-secretase acts only after trimer dissociation or partial disengagement, which are not modeled in this study. Instead, we describe how the W191X mutation perturbs trimeric packing and helix orientation within the preshedding complex, potentially predisposing the system to transient disengagement prior to subsequent proteolytic processing. Given TREM2’s critical role in regulating microglial responses to amyloid plaques, including early stage plaque seeding and clustering,^46,47^ structural disruptions such as W191X may mechanistically link altered microglial function to increased Alzheimer’s disease risk. This work focuses on the transmembrane domains of TREM2–DAP12 using an 80:20 POPC:cholesterol bilayer, which simplifies the complex lipid environment of microglial membranes. The time scales accessible to atomistic MD restrict slower rearrangements, and post-translational modifications are not included. Nevertheless, the observed mutation-specific destabilization patterns are robust across replica and modeling resolutions.

In summary, this study establishes a molecular framework for understanding how C-terminal truncation mutations, such as W191X, destabilize the TREM2–DAP12 complex. It underscores the importance of structural integrity in maintaining signaling-competent receptor assemblies and provides new insights for targeting TREM2 variants in the context of neurodegenerative diseases.

## Methods

### Approach to Build Systems

The TREM2 mutants are made with ColabFold v1.5.2 ^37,48^ using the mutated sequence (structural confidence can be found in Supplemental Figures S19–S25), and the DAP12 dimer is using PDB 2L34.^25^ The complexes are made by merging the TREM2 mutants and DAP12 together as a reference using HADDOCK,^49,50^ AlphaFold2,^37^ AlphaFold3,^38^ and the wild-type complex structure. We simulated a stable conformation. The sequences of each system can be found in Supplemental Table S2. Each system was then equilibrated using all-atom molecular dynamics simulations for 100 ns. The systems utilized in this paper were generated through the use of the CHARMM-GUI Membrane Builder^43^ and the MARTINI Maker^51^ for all-atom (AA) and coarse-grained (CG) simulations, respectively. The protein complex was embedded in a bilayer containing POPC and cholesterol (at a ratio of 80:20), approximating the phosphatidylcholine-rich, cholesterol-moderate composition of neuronal plasma membranes, and has been used in our previous research.^26,52^ This composition also ensures a direct comparison with our previous TREM2–DAP12 simulations. The solvent employed in every simulation was water, and to maintain a neutral charge and a 0.15 M salt concentration, Na^+^ and Cl^–^ ions were added to the system, and protonation states were assigned at pH 7.0 in CHARMM-GUI. The initial configurations for each system are shown in Supplemental Figure S26, while the complete simulation workflow is outlined in Supplemental Figure S27.

### Coarse-Grained Molecular Dynamics Simulations

Coarse-grained simulations of 300 and 200 μs were performed for isoform 1 and isoform 2, respectively, to obtain equilibrated systems embedded in a POPC/cholesterol lipid bilayer. Simulations were carried out using GROMACS version 2019.2.^53^ The MARTINI 2.2P force field^54^ was applied for all systems. Each membrane system underwent energy minimization, followed by equilibration at 310.15 K. Simulations employed a 20 fs integration time step with reaction-field electrostatics (ε_r_ = 15, *r*_coul_ = 1.1 nm) and a Lennard-Jones cutoff of 1.1 nm. Semi-isotropic pressure coupling was applied at 1 bar using the Berendsen barostat during equilibration and the Parrinello–Rahman barostat during production. The MARTINI 2.2P model maps three to five non-hydrogen atoms onto coarse-grained beads, with bead types defined by the atomic composition and chemical characteristics. Beads are categorized into four main classes: charged, polar, nonpolar, and apolar, with five subtypes per class, distinguished by polarity and hydrogen-bonding potential. An overview of the CG simulation setups for various TREM2–DAP12 complexes is provided in Table 2. CG proteins used MARTINI 2.2P with standard MARTINI 2.x secondary-structure restraints on BB beads (helical angles and dihedrals) with no elastic network: helical angle parameters θ_0_ ≈ 96° and *k*_θ_ = 700 kJ mol^–1^ rad^–2^; helical dihedral parameters ϕ_0_ ≈ −120° and *k*_ϕ_ = 400 kJ mol^–1^.

**Table 2:** Details of Each of the Coarse-Grained MD Simulation Systems

Variant	*n*_beads_	*n*_lipids_	*n*_waters_	*n*_ions_	Final box dimensions (nm)
K186A-230	24024	310	6713	152	100.6 × 100.6 × 127.4
K186X-230	19022	310	5075	115	100.2 × 100.2 × 107.0
W194A-230	23904	310	6673	153	100.4 × 100.4 × 127.2
W194X-230	19618	310	5267	118	100.3 × 100.3 × 108.7
WT-219	24875	310	6089	158	100.5 × 100.5 × 130.6
W191A-219	18802	310	4980	116	100.5 × 100.5 × 106.1
W191X-219	24558	320	6883	154	100.7 × 100.7 × 128.7

### All-Atom Molecular Dynamics Simulations

Following 300 or 200 μs of coarse-grained simulations, each system was converted to an all-atom representation using the CHARMM-GUI Martini to All-atom Converter,^42,43^ which implements the backward geometric reconstruction algorithm,^55^ and subsequently simulated for 300 ns. GROMACS version 2020.3 ^53^ was used to perform these AA simulations. The same POPC/cholesterol bilayer composition was retained, with lipids described by the CHARMM36m force field^56,57^ and water represented by the CHARMM-modified TIP3P model.^58^ Each system was first energy-minimized and then equilibrated at 310.15 K and 1 bar, following CHARMM-GUI protocols.^58,59^ All-atom systems were equilibrated for 125 ns before production, following the standard six-step CHARMM-GUI membrane protocol with progressively relaxed restraints.^60^ The equilibration process began with steepest-descent energy minimization, followed by a 125 ps NVT run using the Nosè–Hoover thermostat to maintain a temperature of 310.15 K with a 1 fs time step. This was followed by a 300 ns production simulation in the NPT ensemble, employing the Nosé–Hoover thermostat and the Parrinello–Rahman barostat to sustain the temperature at 310.15 K and pressure at 1 atm.^61,62^ Bond constraints involving hydrogens were applied using the LINCS algorithm.^63^ Periodic boundary conditions were used in all three dimensions. Both the equilibration and production phases used a 1 fs time step. Electrostatic and Lennard-Jones (LJ) interactions were truncated at 12 Å, with a switching function applied to taper LJ interactions to zero, starting from 10 Å. Table 3 summarizes the AA simulation setups for the TREM2–DAP12 complexes. Three independent replicas were conducted. All quantitative analyses were averaged over three independent replicas.

**Table 3:** Details of Each of the All-Atom MD Simulation Systems

Variant	*n*_atoms_	*n*_lipids_	*n*_waters_	*n*_ions_	Final box dimensions (nm)
K186A-230	120584	310	26969	152	97.9 × 97.9 × 130.1
K186X-230	100554	310	20405	115	97.7 × 97.7 × 106.9
W194A-230	120055	310	26793	153	97.4 × 97.4 × 130.7
W194X-230	103026	310	21188	118	96.1 × 96.1 × 113.5
WT-219	123893	310	28060	158	98.3 × 98.3 × 132.0
W191A-219	99717	310	20020	116	99.2 × 99.2 × 103.7
W191X-219	122945	315	27642	154	98.5 × 98.5 × 130.3

### Simulation Analysis Techniques

All simulations were analyzed using in-house Python (3.10) scripts built primarily on the MDAnalysis package.^64,65^ Visualizations were generated using Matplotlib,^66^ and trajectory inspection was carried out with VMD 1.9.3.^67^

#### Hydrogen Bond Analysis

Hydrogen bond probabilities were calculated using the Hydrogen Bond Analysis module in MDAnalysis^68^ via custom Python scripts. A donor–acceptor distance cutoff of 3 Å and a donor–hydrogen–acceptor angle cutoff of 150° were applied.

#### π–π Stacking Analysis

π–π stacking interactions were evaluated using an in-house Python script based on PySoftK.^69^ Ring centroids were computed as the mean position of ring atoms, and ring plane orientation was defined using three noncollinear atoms. Interactions were classified using a centroid distance cutoff of 6.5 Å^70^ and an interplanar angle criterion: ≤ 30° for parallel-displaced and 60–120° for T-shaped stacking.

#### Hydrophobic Contacts

Hydrophobic contacts were defined as any side-chain carbon–carbon pair (backbone N, Cα, C, and O excluded) with a minimum interatomic distance ≤4.5 Å.

#### Salt Bridges

Salt bridges were defined by a minimum N···O distance of ≤3.2 Å between basic groups (Lys NZ, Arg NH1/NH2/NE, protonated His ND1/NE2, or N-terminal N) and acidic groups (Asp OD1/OD2, Glu OE1/OE2, or C-terminal O/OXT).

#### *Z*-Axis Positioning

The *z*-axis positioning was calculated from all-atom trajectories by using custom Python scripts with MDAnalysis. It was defined as the center-of-mass (COM) distance of each transmembrane helix projected onto the membrane.

#### Conformational Clustering of TREM2–DAP12 Complexes

To identify stable conformational states of the TREM2–DAP12 complexes from coarse-grained simulations, we first extracted interresidue distance features from backbone beads (BBs) spaced every five residues along the transmembrane helices of TREM2 and both DAP12 chains. At each simulation frame, we computed pairwise distance matrices between the selected BB atoms of TREM2 and those of each DAP12 chain independently using MDAnalysis. These matrices were flattened into feature vectors and concatenated across frames to form a high-dimensional data set representing the dynamic spatial organization of the transmembrane helices.

We then applied UMAP for dimensionality reduction, followed by clustering with HDBSCAN. UMAP and HDBSCAN parameters were kept fixed within each isoform to ensure reproducibility. The selected values fall within standard, physically reasonable ranges commonly used for molecular dynamics trajectory embeddings. The resulting low-dimensional embeddings captured dominant structural states sampled by each system and were used to initialize subsequent all-atom simulations. Previously, we have used this general approach to characterizing the conformations taken by antimicrobial peptides,^71^ apo-c3,^72^ interacting lipids,^73^ and polymers.^52,74–76^ For isoform 1, UMAP was performed with n_neighbors set to 50, and subsequent clustering used HDBSCAN with min_cluster_size = 50 and cluster_selection_epsilon = 2.0. For isoform 2, the same min_cluster_size was used, with n_neighbors = 10 and cluster_selection_epsilon = 1.5.

#### Contact Map Analysis

Residue-level contact maps between TREM2 and DAP12 were calculated by using a custom Python script with MDAnalysis. Contacts were defined as any pair of heavy atoms from TREM2 and DAP12 within 3 Å. At each frame, a binary contact matrix was generated and accumulated over the trajectory. The final contact map represents the frequency of residue–residue contacts over time, enabling the identification of stable interaction interfaces.

#### Root Mean Square Fluctuation (RMSF) Analysis

RMSF was calculated using a custom Python script with MDAnalysis and NumPy. α Carbon atoms were selected, and the trajectory was iterated to compute the mean atomic positions and displacements over time. The RMSF for each residue was computed as

$${\mathrm{RMSF}}_{i}=\sqrt{\frac{1}{T}\overset{T}{\underset{t=1}{\sum }}{∥{r⃗}_{i}(t)-\langle {r⃗}_{i}\rangle ∥}^{2}}$$

where *r⃗*_*i*_(*t*) is the position of atom *i* at frame *t* and ⟨*r⃗*_*i*_⟩ is its time-averaged position. This analysis was performed across the full trajectory for each system to assess the per-residue flexibility.

## Supplementary Material



## Data Availability

Example trajectories and system setup for analysis are available in a Zenodo repository, accessible at 10.5281/zenodo.15746936. The code used for the simulations is available on the GitHub repository, accessible at https://github.com/Lorenz-Lab-KCL/publications/tree/main/2025/TREM2_DAP12_Mutations.

## Notes

Sequence alignment of TREM2 isoforms (Figure S1); structural ensembles and TREM2–DAP12 contact-frequency maps for each variant (Figures S2–S8); contact-frequency maps across replicates/clusters (Figures S9–S15); hydrogen-bond occupancies for K186–D50 (Figures S16–S17); center-of-mass distance analyses quantifying interface disruption (Figure S18); ColabFold/AlphaFold model-confidence diagnostics for all variants (Figures S19–S25); initial complex models and complete simulation workflow (Figures S26–S27); backbone RMSD traces (Figure S28); helix-tilt time series and distributions (Figures S29–S30); overlaid interfacial contact pairs across mutants (Figure S31); replicate RMSF profiles aligned to DAP12 for both isoforms (Figures S32–S33); persistent residue–residue contact tables (Table S1); sequences used in simulations (Table S2); and brief analysis methods for RMSD and tilt-angle calculations (PDF)

## References

[1] Jonsson T., Stefansson H., Steinberg S., Jonsdottir I., Jonsson P. V., Snaedal J., Bjornsson S., Huttenlocher J., Levey A. I., Lah J. J. (2013). Variant
of TREM2 associated with the risk of Alzheimer’s disease. N. Engl. J. Med..

[2] Huang Y., Mucke L. (2012). Alzheimer Mechanisms and Therapeutic Strategies. Cell.

[3] D’Andrea M. R., Cole G. M., Ard M. D. (2004). The microglial phagocytic role with
specific plaque types in the Alzheimer disease brain. Neurobiol. Aging..

[4] Dickson D. W. (1999). Microglia
in Alzheimer’s disease and transgenic models: How close the
fit?. Am. J. Pathol..

[5] Guerreiro R., Wojtas A., Bras J., Carrasquillo M., Rogaeva E., Majounie E., Cruchaga C., Sassi C., Kauwe J. S., Younkin S. (2013). TREM2
variants in Alzheimer’s
disease. N. Engl. J. Med..

[6] Colonna M. (2023). The biology
of TREM receptors. Nat. Rev. Immunol..

[7] Ulland T. K., Colonna M. (2018). TREM2-a key player
in microglial biology and Alzheimer
disease. Nat. Rev. Neurol..

[8] Wang Y., Cella M., Mallinson K., Ulrich J. D., Young K. L., Robinette M. L., Gilfillan S., Krishnan G. M., Sudhakar S., Zinselmeyer B. H. (2015). TREM2 lipid sensing sustains the microglial
response in an Alzheimer’s disease model. Cell.

[9] Cannon J. P., O’Driscoll M., Litman G. W. (2012). Specific lipid recognition is a general
feature of CD300 and TREM molecules. Immunogenetics.

[10] Daws M. R., Sullam P. M., Niemi E. C., Chen T. T., Tchao N. K., Seaman W. E. (2003). Pattern recognition
by TREM-2: Binding of anionic ligands. J. Immunol..

[11] Kawabori M., Kacimi R., Kauppinen T., Calosing C., Kim J. Y., Hsieh C. L., Nakamura M. C., Yenari M. A. (2015). Triggering receptor
expressed on myeloid cells 2 (TREM2) deficiency attenuates phagocytic
activities of microglia and exacerbates ischemic damage in experimental
stroke. J. Neurosci..

[12] Kober D. L., Brett T. J. (2017). TREM2-ligand interactions
in health and disease. J. Mol. Biol..

[13] Ulland T. K., Song W. M., Huang S.-C.-C., Ulrich J. D., Sergushichev A., Beatty W. L., Loboda A. A., Zhou Y., Cairns N. J., Kambal A. (2017). TREM2
maintains microglial metabolic fitness in Alzheimer’s
disease. Cell.

[14] Zhou R., Yang G., Guo X., Zhou Q., Lei J., Shi Y. (2019). Recognition of the
amyloid precursor protein by human *γ*-secretase. Science.

[15] Thornton P., Sevalle J., Deery M. J., Fraser G., Zhou Y., Ståhl S., Franssen E. H., Dodd R. B., Qamar S., Gomez Perez-Nievas B. (2017). TREM 2 shedding by cleavage at the H157-S158
bond is accelerated for the Alzheimer’s disease-associated
H157Y variant. EMBO Mol. Med..

[16] Feuerbach D., Schindler P., Barske C., Joller S., Beng-Louka E., Worringer K. A., Kommineni S., Kaykas A., Ho D. J., Ye C. (2017). ADAM17 is the main sheddase for the generation of human
triggering receptor expressed in myeloid cells (hTREM2) ectodomain
and cleaves TREM2 after Histidine 157. Neurosci.
Lett..

[17] Steiner A., Schlepckow K., Brunner B., Steiner H., Haass C., Hagn F. (2020). *γ*-Secretase cleavage of the Alzheimer risk
factor TREM 2 is determined by its intrinsic structural dynamics. EMBO J..

[18] Hamerman J. A., Jarjoura J. R., Humphrey M. B., Nakamura M. C., Seaman W. E., Lanier L. L. (2006). Cutting edge: Inhibition of TLR and
FcR responses in
macrophages by triggering receptor expressed on myeloid cells (TREM)-2
and DAP12. J. Immunol..

[19] Peng Q., Malhotra S., Torchia J. A., Kerr W. G., Coggeshall K. M., Humphrey M. B. (2010). TREM2- and DAP12-Dependent
Activation of PI3K Requires
DAP10 and Is Inhibited by SHIP1. Sci. Signal..

[20] Kleinberger G., Yamanishi Y., Suárez-Calvet M., Czirr E., Lohmann E., Cuyvers E., Struyfs H., Pettkus N., Wenninger-Weinzierl A., Mazaheri F. (2014). TREM2 mutations implicated
in neurodegeneration impair cell surface transport and phagocytosis. Sci. Transl. Med..

[21] Guerreiro R., Bilgic B., Guven G., Brás J., Rohrer J., Lohmann E., Hanagasi H., Gurvit H., Emre M. (2013). A novel compound heterozygous mutation in TREM2 found in a Turkish
frontotemporal dementia-like family. Neurobiol.
Aging..

[22] Paloneva J., Manninen T., Christman G., Hovanes K., Mandelin J., Adolfsson R., Bianchin M., Bird T., Miranda R., Salmaggi A. (2002). Mutations in two genes encoding different subunits
of a receptor signaling complex result in an identical disease phenotype. Am. J. Hum. Genet..

[23] Swain P. S., Panda S., Pati S., Dehury B. (2023). Computational saturation
mutagenesis to explore the effect of pathogenic mutations on extra-cellular
domains of TREM2 associated with Alzheimer’s and Nasu-Hakola
disease. J. Mol. Model..

[24] Mishra S., Swain P. S., Pati S., Dehury B. (2025). Extracellular domain
of TREM2 possess two distinct ligand recognition sites: Insights from
machine-learning guided docking and all-atoms molecular dynamics simulations. Heliyon.

[25] Call M. E., Wucherpfennig K. W., Chou J. J. (2010). The structural basis for intramembrane
assembly of an activating immunoreceptor complex. Nat. Immunol..

[26] Zhong Z., Ulmschneider M. B., Lorenz C. D. (2024). Unraveling the Molecular Dance: Insights
into TREM2/DAP12 Complex Formation in Alzheimer’s Disease through
Molecular Dynamics Simulations. ACS Omega.

[27] Jin S. C., Carrasquillo M. M., Benitez B. A., Skorupa T., Carrell D., Patel D., Lincoln S., Krishnan S., Kachadoorian M., Reitz C. (2015). TREM2 is associated with increased risk for Alzheimer’s
disease in African Americans. Mol. Neurodegener..

[28] Moutinho M., Coronel I., Tsai A. P., Di Prisco G. V., Pennington T., Atwood B. K., Puntambekar S. S., Smith D. C., Martinez P., Han S. (2023). TREM2
splice isoforms generate soluble TREM2 species that disrupt long-term
potentiation. Genome Med..

[29] Bharadwaj S., Groza Y., Mierzwicka J. M., Malý P. (2024). Current understanding
on TREM-2 molecular biology and physiopathological functions. Int. Immunopharmacol..

[30] Giraldo M., Lopera F., Siniard A. L., Corneveaux J. J., Schrauwen I., Carvajal J., Muñoz C., Ramirez-Restrepo M., Gaiteri C., Myers A. J. (2013). Variants
in triggering receptor expressed on myeloid cells 2 are associated
with both behavioral variant frontotemporal lobar degeneration and
Alzheimer’s disease. Neurobiol. Aging.

[31] Celarain N., Sánchez-Ruiz de
Gordoa J., Zelaya M. V., Roldán M., Larumbe R., Pulido L., Echavarri C., Mendioroz M. (2016). TREM2 upregulation correlates with 5-hydroxymethycytosine
enrichment in Alzheimer’s disease hippocampus. Clinical Epigenetics.

[32] Ma L., Allen M., Sakae N., Ertekin-Taner N., Graff-Radford N. R., Dickson D. W., Younkin S. G., Sevlever D. (2016). Expression
and processing analyses of wild type and p.R47H TREM2 variant in Alzheimer’s
disease brains. Mol. Neurodegener..

[33] Jin S. C., Benitez B. A., Karch C. M., Cooper B., Skorupa T., Carrell D., Norton J. B., Hsu S., Harari O., Cai Y. (2014). Coding variants in TREM2
increase risk for Alzheimer’s
disease. Hum. Mol. Genet..

[34] Callaway E. (2024). These’movies’
of proteins in action are revealing the hidden biology of cells. Nature.

[35] Shoemaker S. C., Ando N. (2018). X-rays in the cryo-electron microscopy
era: Structural biology’s
dynamic future. Biochemistry.

[36] Beck M., Covino R., Hänelt I., Müller-McNicoll M. (2024). Understanding
the cell: Future views of structural biology. Cell.

[37] Jumper J., Evans R., Pritzel A., Green T., Figurnov M., Ronneberger O., Tunyasuvunakool K., Bates R., Žídek A., Potapenko A. (2021). Highly accurate protein structure prediction
with AlphaFold. Nature.

[38] Abramson J., Adler J., Dunger J., Evans R., Green T., Pritzel A., Ronneberger O., Willmore L., Ballard A. J., Bambrick J. (2024). Accurate
structure prediction of biomolecular
interactions with AlphaFold 3. Nature.

[39] Malhotra Y., John J., Yadav D., Sharma D., Rawal K., Mishra V., Chaturvedi N. (2025). Advancements in protein
structure prediction: A comparative overview of AlphaFold and its
derivatives. Comput. Biol. Med..

[40] Karplus M., McCammon J. A. (2002). Molecular dynamics
simulations of biomolecules. Nat. Struct. Biol..

[41] Hospital A., Goñi J. R., Orozco M., Gelpí J. L. (2015). Molecular
dynamics simulations: Advances and applications. Adv. Appl. Bioinforma. Chem..

[42] Qi Y., Cheng X., Han W., Jo S., Schulten K., Im W. (2014). CHARMM-GUI PACE CG
Builder for solution, micelle, and bilayer coarse-grained
simulations. J. Chem. Inf. Model..

[43] Jo S., Kim T., Iyer V. G., Im W. (2008). CHARMM-GUI: A web-based graphical
user interface for CHARMM. J. Comput. Chem..

[44] Wang S., Sudan R., Peng V., Zhou Y., Du S., Yuede C. M., Lei T., Hou J., Cai Z., Cella M. (2022). TREM2 drives microglia
response to amyloid-*β* via SYK-dependent and-independent
pathways. Cell.

[45] Zhang L., Xiang X., Li Y., Bu G., Chen X.-F. (2025). TREM2 and
sTREM2 in Alzheimer’s disease: From mechanisms to therapies. Mol. Neurodegener..

[46] Parhizkar S., Arzberger T., Brendel M., Kleinberger G., Deussing M., Focke C., Nuscher B., Xiong M., Ghasemigharagoz A., Katzmarski N. (2019). Loss of TREM2 function
increases amyloid seeding but reduces plaque-associated ApoE. Nat. Neurosci..

[47] Ulrich J. D., Ulland T. K., Colonna M., Holtzman D. M. (2017). Elucidating
the
role of TREM2 in Alzheimer’s disease. Neuron.

[48] Mirdita M., Schütze K., Moriwaki Y., Heo L., Ovchinnikov S., Steinegger M. (2022). ColabFold: Making protein folding accessible to all. Nat. Methods.

[49] Dominguez C., Boelens R., Bonvin A. M. (2003). HADDOCK: A protein-
protein docking
approach based on biochemical or biophysical information. J. Am. Chem. Soc..

[50] Van
Zundert G., Rodrigues J., Trellet M., Schmitz C., Kastritis P., Karaca E., Melquiond A., van Dijk M., De Vries S., Bonvin A. (2016). The HADDOCK2. 2 web
server: User-friendly integrative modeling of biomolecular complexes. J. Mol. Biol..

[51] Siewert J. M., Risselada H.J., Yefimov S., Tieleman D.P., De Vries A.H. (2007). The MARTINI
force field: Coarse grained model for biomolecular simulations. J. Phys. Chem. B.

[52] López-Ríos
de Castro R., Ziolek R. M., Lorenz C. D. (2023). Topology-controlled
self-assembly of amphiphilic block copolymers. Nanoscale.

[53] Van
Der Spoel D., Lindahl E., Hess B., Groenhof G., Mark A. E., Berendsen H. J. (2005). GROMACS: Fast, flexible, and free. J. Comput. Chem..

[54] Qi Y., Ingólfsson H. I., Cheng X., Lee J., Marrink S. J., Im W. (2015). CHARMM-GUI martini maker for coarse-grained simulations with the
martini force field. J. Chem. Theory Comput..

[55] Wassenaar T. A., Pluhackova K., Bockmann R. A., Marrink S. J., Tieleman D. P. (2014). Going backward:
A flexible geometric approach to reverse transformation from coarse
grained to atomistic models. J. Chem. Theory
Comput..

[56] Huang J., Rauscher S., Nawrocki G., Ran T., Feig M., De Groot B. L., Grubmüller H., MacKerell Jr A. D. (2017). CHARMM36m:
An improved force field for folded and intrinsically disordered proteins. Nat. Methods.

[57] Klauda J. B., Venable R. M., Freites J. A., O’Connor J. W., Tobias D. J., Mondragon-Ramirez C., Vorobyov I., MacKerell
Jr A. D., Pastor R. W. (2010). Update of the CHARMM all-atom additive
force field for lipids: Validation on six lipid types. J. Phys. Chem. B.

[58] MacKerell
Jr A. D., Bashford D., Bellott M., Dunbrack
Jr R. L., Evanseck J. D., Field M. J., Fischer S., Gao J., Guo H., Ha S. (1998). All-atom empirical potential
for molecular modeling and dynamics studies of proteins. J. Phys. Chem. B.

[59] Best R. B., Zhu X., Shim J., Lopes P. E., Mittal J., Feig M., MacKerell Jr A. D. (2012). Optimization of the additive CHARMM all-atom protein
force field targeting improved sampling of the backbone *ϕ*, *ψ* and side-chain *χ*1 and *χ*2 dihedral angles. J. Chem. Theory Comput..

[60] Lee J., Cheng X., Jo S., MacKerell A. D., Klauda J. B., Im W. (2016). CHARMM-GUI input generator for NAMD,
GROMACS, AMBER, OpenMM, and CHARMM/OpenMM simulations using the CHARMM36
additive force field. Biophys. J..

[61] Nosé S. U. I. (2002). A molecular
dynamics method for simulations in the canonical ensemble. Mol. Phys..

[62] Parrinello M., Rahman A. (1981). Polymorphic transitions in single
crystals: A new molecular
dynamics method. J. Appl. Phys..

[63] Hess B., Bekker H., Berendsen H. J., Fraaije J. G. (1997). LINCS: A linear
constraint solver for molecular simulations. J. Comput. Chem..

[64] Gowers, R. J. ; Linke, M. ; Barnoud, J. ; Reddy, T. J. ; Melo, M. N. ; Seyler, S. L. ; Domanski, J. ; Dotson, D. L. ; Buchoux, S. ; Kenney, I. M. MDAnalysis: A Python package for the rapid analysis of molecular dynamics simulations Proceedings Of The 15th Python In Science Conference OSTI 2016 105

[65] Michaud-Agrawal N., Denning E. J., Woolf T. B., Beckstein O. (2011). MDAnalysis:
A toolkit for the analysis of molecular dynamics simulations. J. Comput. Chem..

[66] Hunter J. D. (2007). Matplotlib:
A 2D graphics environment. Comput. Sci. Eng..

[67] Humphrey W., Dalke A., Schulten K. (1996). VMD: Visual
molecular dynamics. J. Mol. Graphics..

[68] Smith P., Ziolek R. M., Gazzarrini E., Owen D. M., Lorenz C. D. (2019). On the
interaction of hyaluronic acid with synovial fluid lipid membranes. Phys. Chem. Chem. Phys..

[69] López-Ríos
de Castro R., Santana-Bonilla A., Ziolek R. M., Lorenz C. D. (2025). Automated
analysis of soft matter interfaces, interactions, and self-assembly
with PySoftK. J.Chem. Inf. Model.

[70] Piovesan D., Minervini G., Tosatto S. C. (2016). The RING 2.0 web server for high
quality residue interaction networks. Nucleic
Acids Res..

[71] Serian M., Mason A. J., Lorenz C. D. (2024). Emergent
conformational and aggregation
properties of synergistic antimicrobial peptide combinations. Nanoscale.

[72] Al
Badri M. A., Smith P., Al Jamal K. T., Lorenz C. D. (2022). Nanomaterial
functionalization modulates hard protein corona formation: Atomistic
simulations applied to graphitic materials. Adv. Mater. Interfaces.

[73] Smith P., Quinn P. J., Lorenz C. D. (2020). Two coexisting membrane structures
are defined by lateral and transbilayer interactions between sphingomyelin
and cholesterol. Langmuir.

[74] Ziolek R. M., Smith P., Pink D. L., Dreiss C. A., Lorenz C. D. (2021). Unsupervised
learning unravels the structure of four-arm and linear block copolymer
micelles. Macromolecules.

[75] Ziolek R. M., Santana-Bonilla A., López-Ríos de Castro R., Kuhn R., Green M., Lorenz C. D. (2022). Conformational heterogeneity
and interchain percolation revealed in an amorphous conjugated polymer. ACS Nano.

[76] López-Ríosde
Castro R., Ziolek R. M., Ulmschneider M. B., Lorenz C. D. (2024). Therapeutic peptides are preferentially solubilized
in specific microenvironments within PEG-PLGA polymer nanoparticles. Nano Lett..

[77] King’s Computational Research, Engineering and Technology Environment (CREATE). eResearch; King’s College London, 2025. 10.18742/rnvf-m076.

