# Supplementary material for: Mechanistic Disruption of the TREM2–DAP12 Transmembrane Complex by Alzheimer’s Disease Mutations: A Multiscale Simulation Study
Source: J Chem Inf Model. 2025 Nov 26;65(24):13315–27. doi: 10.1021/acs.jcim.5c01891 (PMC12728928; doi:10.1021/acs.jcim.5c01891)
Supplement: Supplementary file 1 [file ci5c01891_si_001.pdf]

Supporting Information for "Mechanistic Disruption of the TREM2–DAP12 Transmembrane Complex by Alzheimer's Disease Mutations: A Multiscale Simulation Study"

Zhiwen Zhong,[†,‡] Martin B. Ulmschneider,[‡] and Christian D. Lorenz[*,¶]

†*Department of Physics, King's College London, London, WC2R 2LS, UK*

‡*Department of Chemistry, King's College London, London, SE1 1DB, UK*

¶*Department of Engineering, King'sCollege London, London WC2R 2LS, UK*

E-mail: chris.lorenz@kcl.ac.uk

This PDF file includes:

Figure S1 to S32

Tables S1 to S2

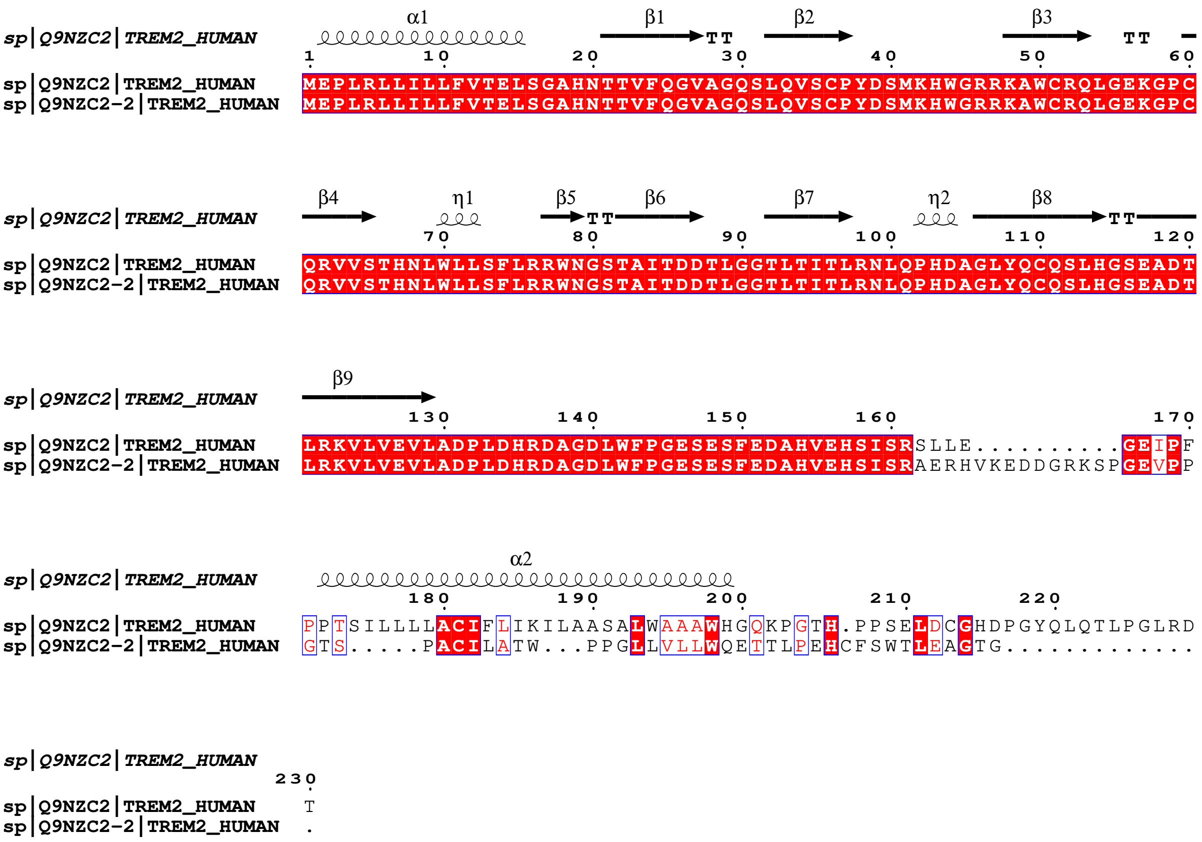

Figure S1: Sequence alignment of two TREM2 isoforms. Isoform 1 (Q9NZC2) corresponds to the full-length 230-amino-acid sequence, while isoform 2 (Q9NZC2-2) is a truncated variant consisting of 219 residues. Secondary structure annotations for isoform 1 were predicted using AlphaFold2 and are indicated above the sequences. The alignment highlights the conserved regions and structural differences between the two isoforms.

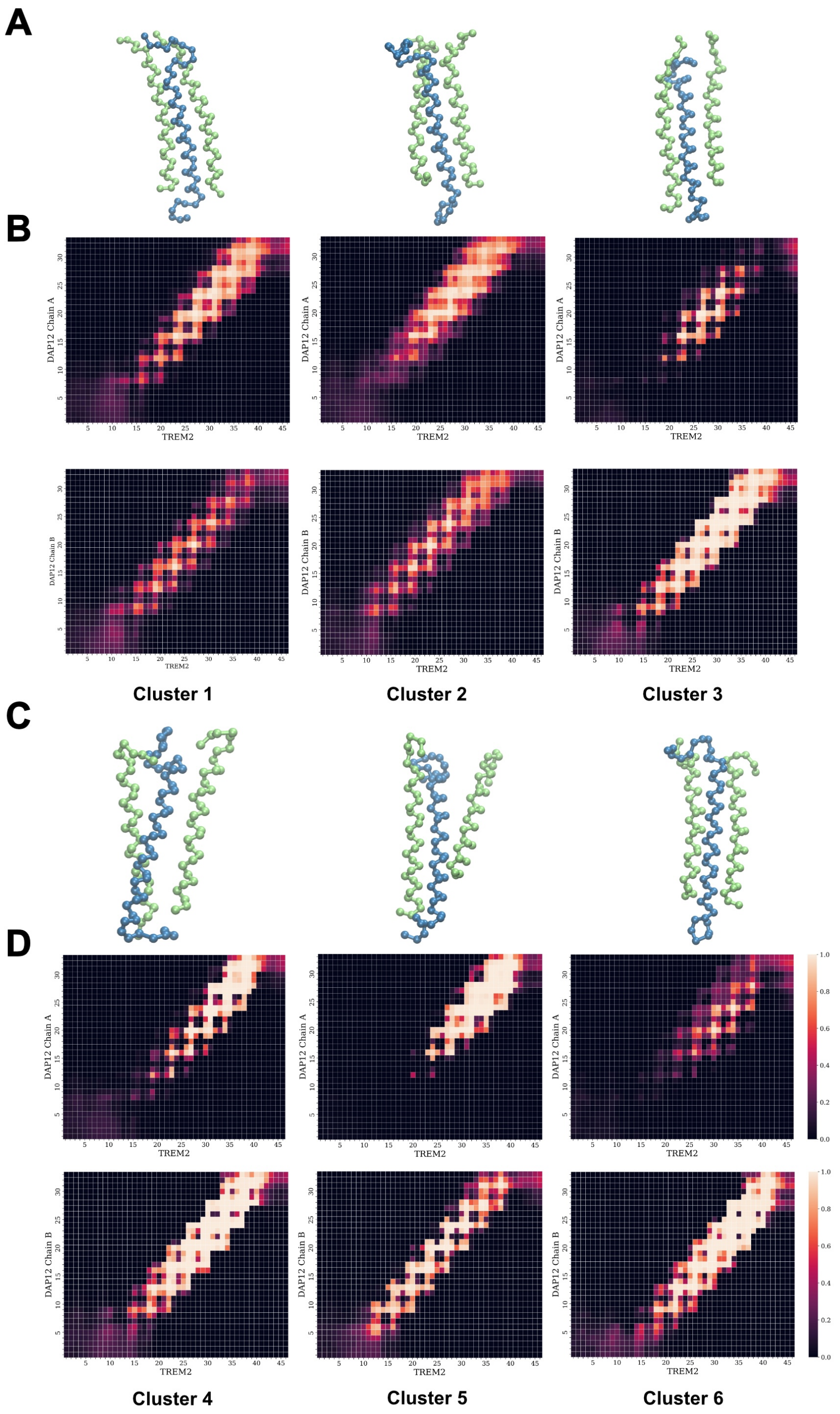

Figure S2: Structural ensembles and contact frequency maps of the K186A-230 system. (A) Representative transmembrane conformations of Cluster 1, Cluster 2, and Cluster 3 from the K186A-230 system, extracted from the clusters based on distance-based clustering of MD trajectories. (B) Corresponding contact maps showing contacts between TREM2 and DAP12 Chain A and Chain B for Clusters 1-3. Contact frequency is color-coded, with lighter shades indicating higher persistence. (C) Representative transmembrane conformations of Cluster 4, Cluster 5, and Cluster 6. (D) Contact frequency matrices for Clusters 4-6, presented as in panel B. The matrices reflect stable inter-residue interactions that define the unique packing arrangements of each cluster.

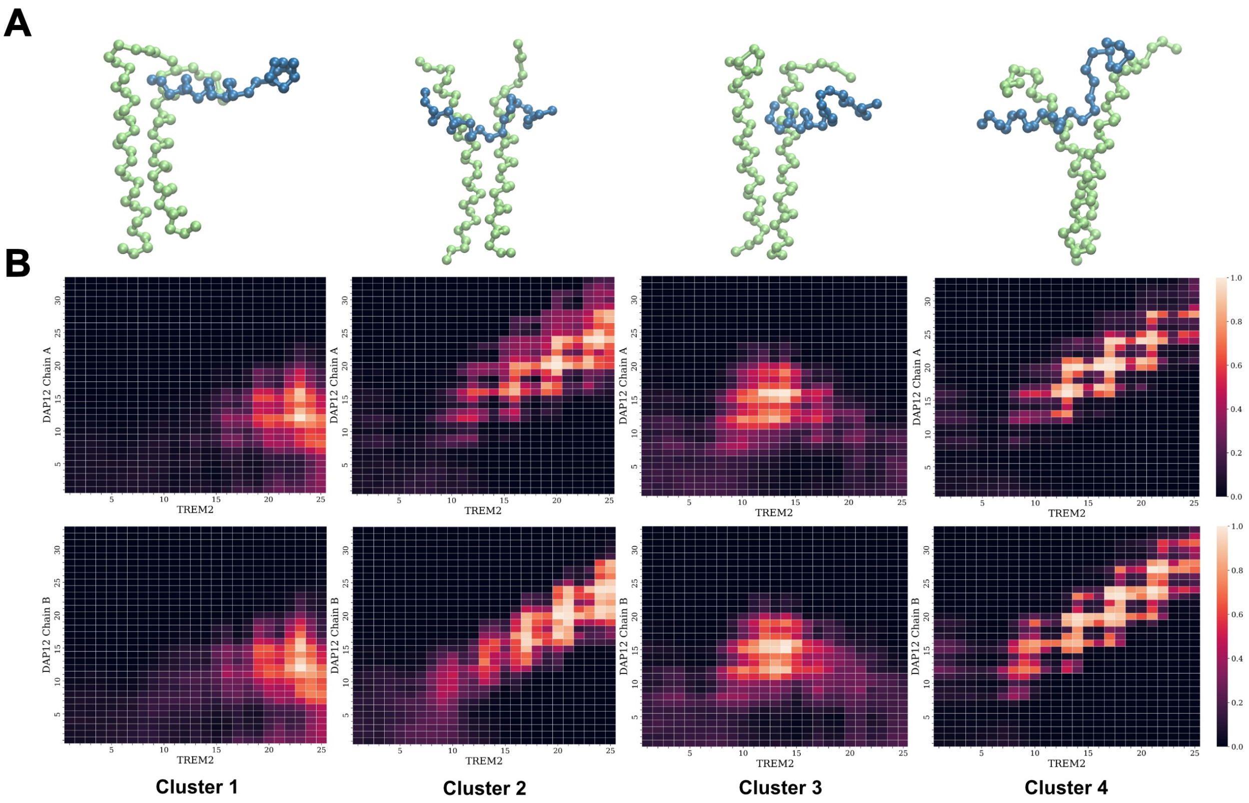

Figure S3: Structural ensembles and contact frequency maps of the K186X-230 system. (A) Representative transmembrane conformations of Cluster 1 to Cluster 4, derived from distance-based clustering of MD trajectories. The structural models illustrate diverse orientations and packing arrangements of the TREM2-DAP12 complex in the absence of K186. (B) Corresponding contact frequency maps between TREM2 and DAP12 Chain A and Chain B for Clusters 1-4. Contact frequency is colour-coded from low (dark) to high (light), highlighting distinct interaction patterns that characterise each conformational state.

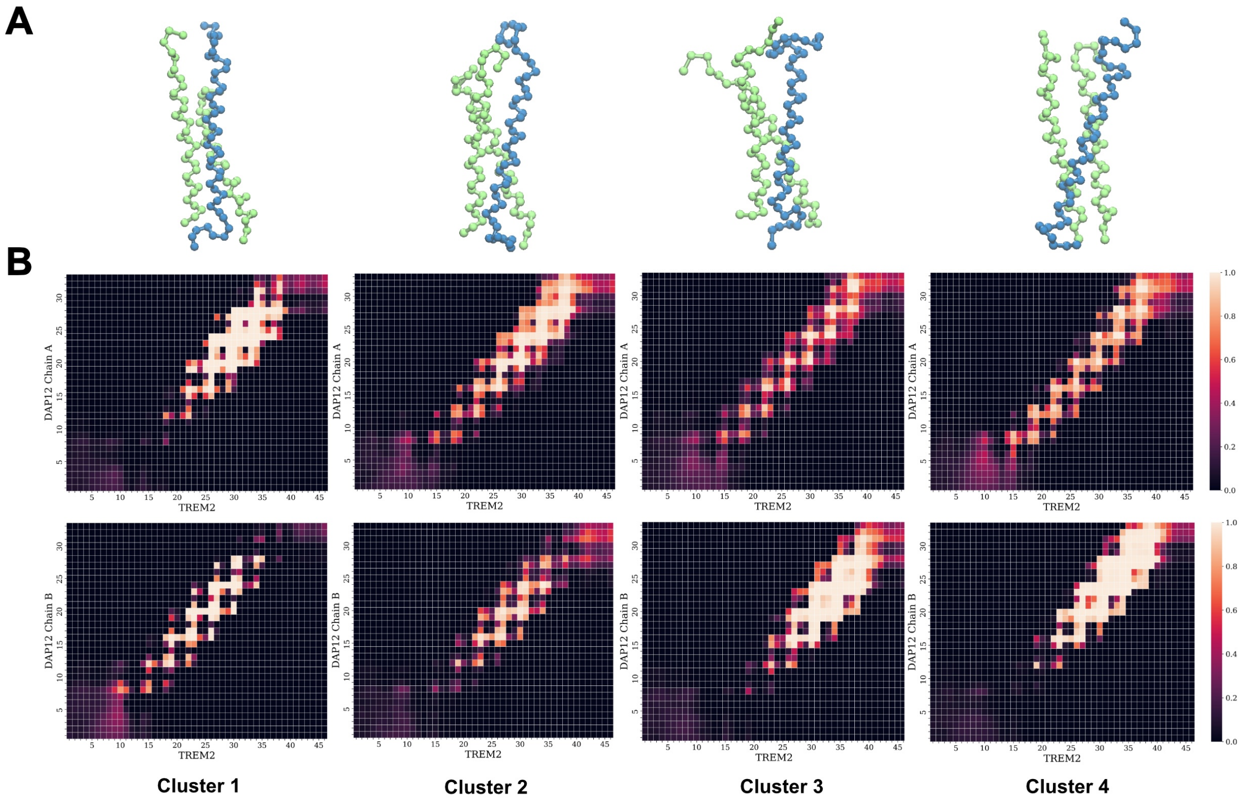

Figure S4: Structural ensembles and contact frequency maps of the W194A-230 system. (A) Representative transmembrane conformations of Cluster 1 to Cluster 4, obtained through distance-based clustering of molecular dynamics trajectories. The structural models display varied spatial arrangements of the TREM2-DAP12 complex in the absence of the W194 residue, illustrating shifts in helix packing and orientation. (B) Corresponding contact frequency maps between TREM2 and DAP12 Chain A and Chain B for Clusters 1-4. Frequency values range from low (black) to high (white), with intermediate contacts shown in shades of red.

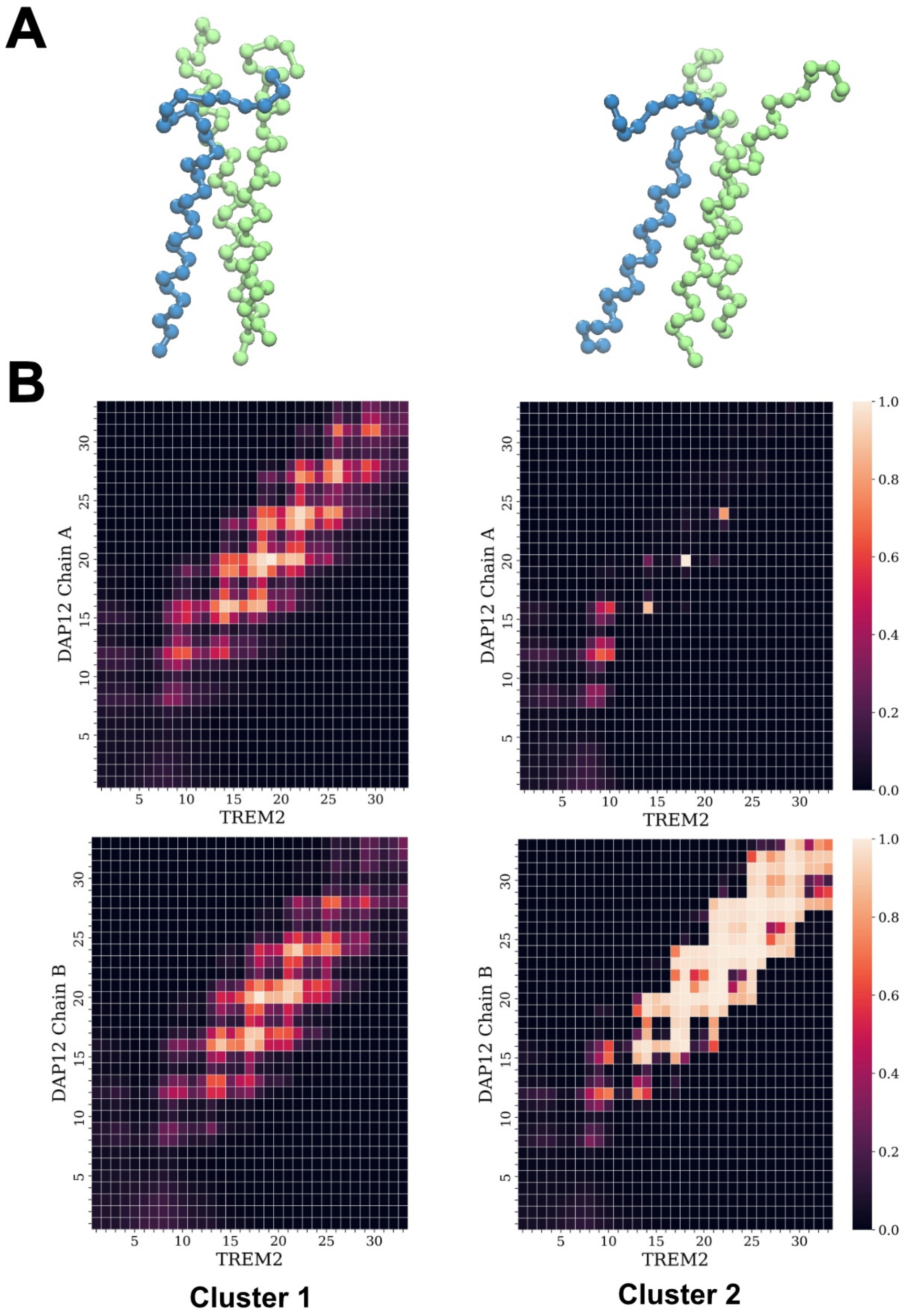

Figure S5: Structural ensembles and contact frequency maps of the W194X-230 system. (A) Representative transmembrane conformations of Cluster 1 and Cluster 2, identified through distance-based clustering of MD trajectories. The snapshots highlight altered packing geometries between TREM2 and DAP12 in the absence of the W194 residue, particularly in helix-helix interface positioning. (B) Corresponding contact frequency maps between TREM2 and DAP12 Chain A and Chain B for Clusters 1 and 2. Contact frequencies are colour-coded from low (black) to high (white), revealing asymmetric and cluster-specific interaction patterns. Notably, Cluster 2 shows significantly reduced contacts with Chain A and compensatory enhancement with Chain B.

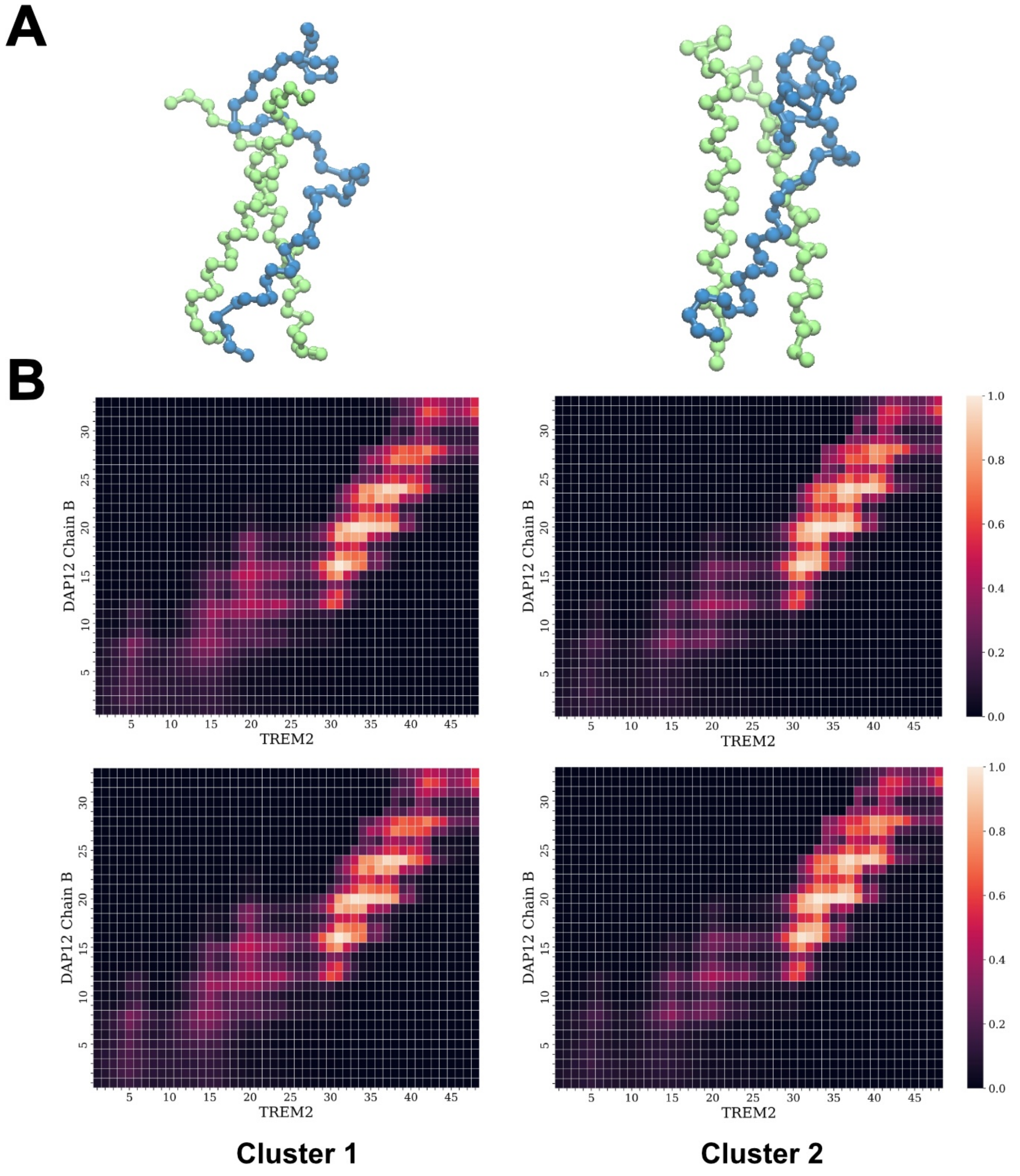

Figure S6: Structural ensembles and contact frequency maps of the WT-219 system. (A) Representative transmembrane conformations of Cluster 1 and Cluster 2, derived from distance-based clustering of MD trajectories. (B) Corresponding contact frequency maps between TREM2 and DAP12 Chain A and Chain B for Clusters 1 and 2. Contact frequencies are color-coded from low (black) to high (white), highlighting consistent and symmetric interaction patterns across both DAP12 chains. Compared to Cluster 1, Cluster 2 exhibits reduced contact frequency in the central transmembrane region.

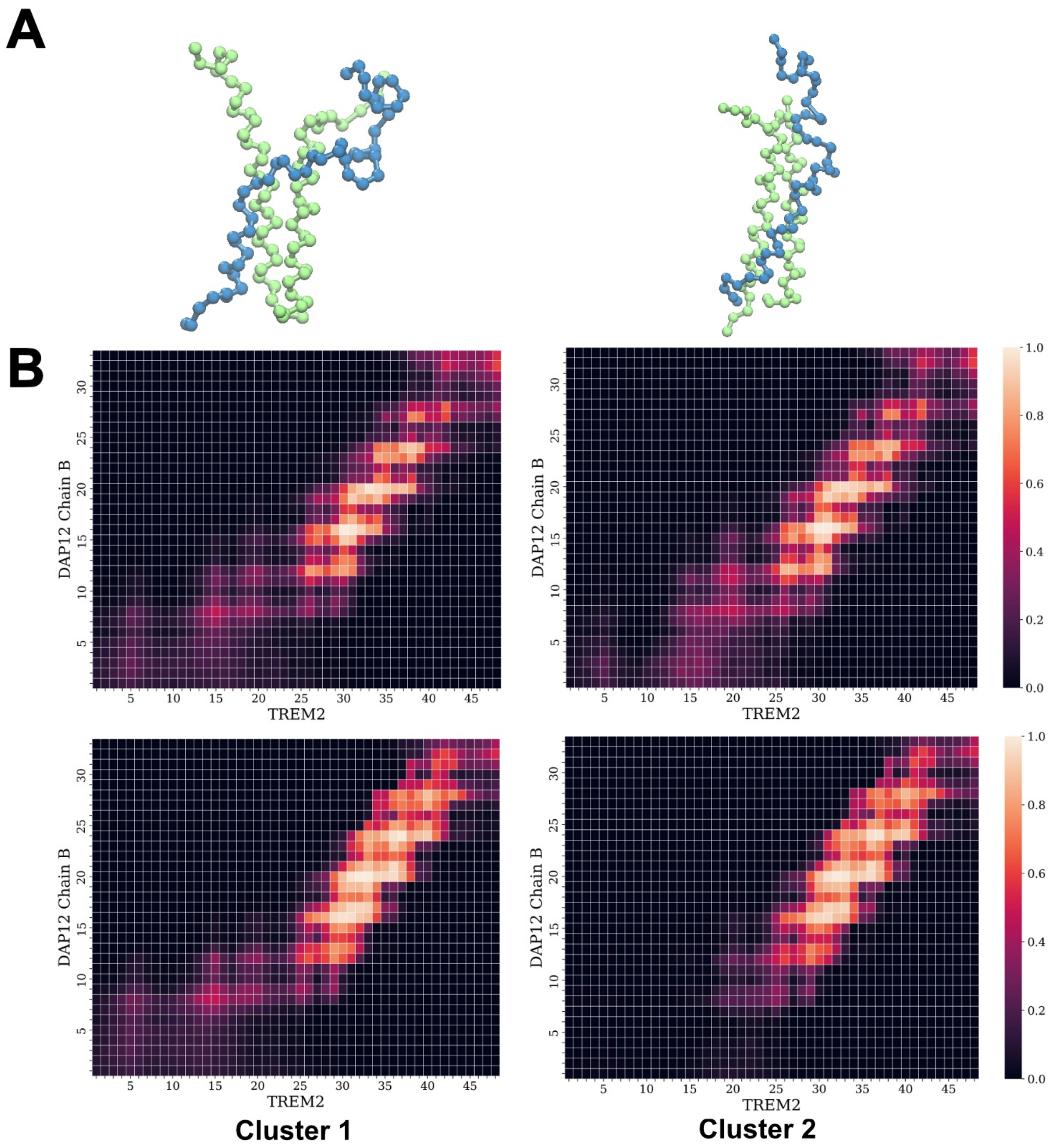

Figure S7: Structural ensembles and contact frequency maps of the W191A-219 system. (A) Representative transmembrane conformations of Cluster 1 and Cluster 2 derived from MD trajectory clustering. (B) Corresponding contact frequency maps between TREM2 and DAP12 Chain A and Chain B for each cluster. Color scale denotes contact frequency from low (black) to high (white).

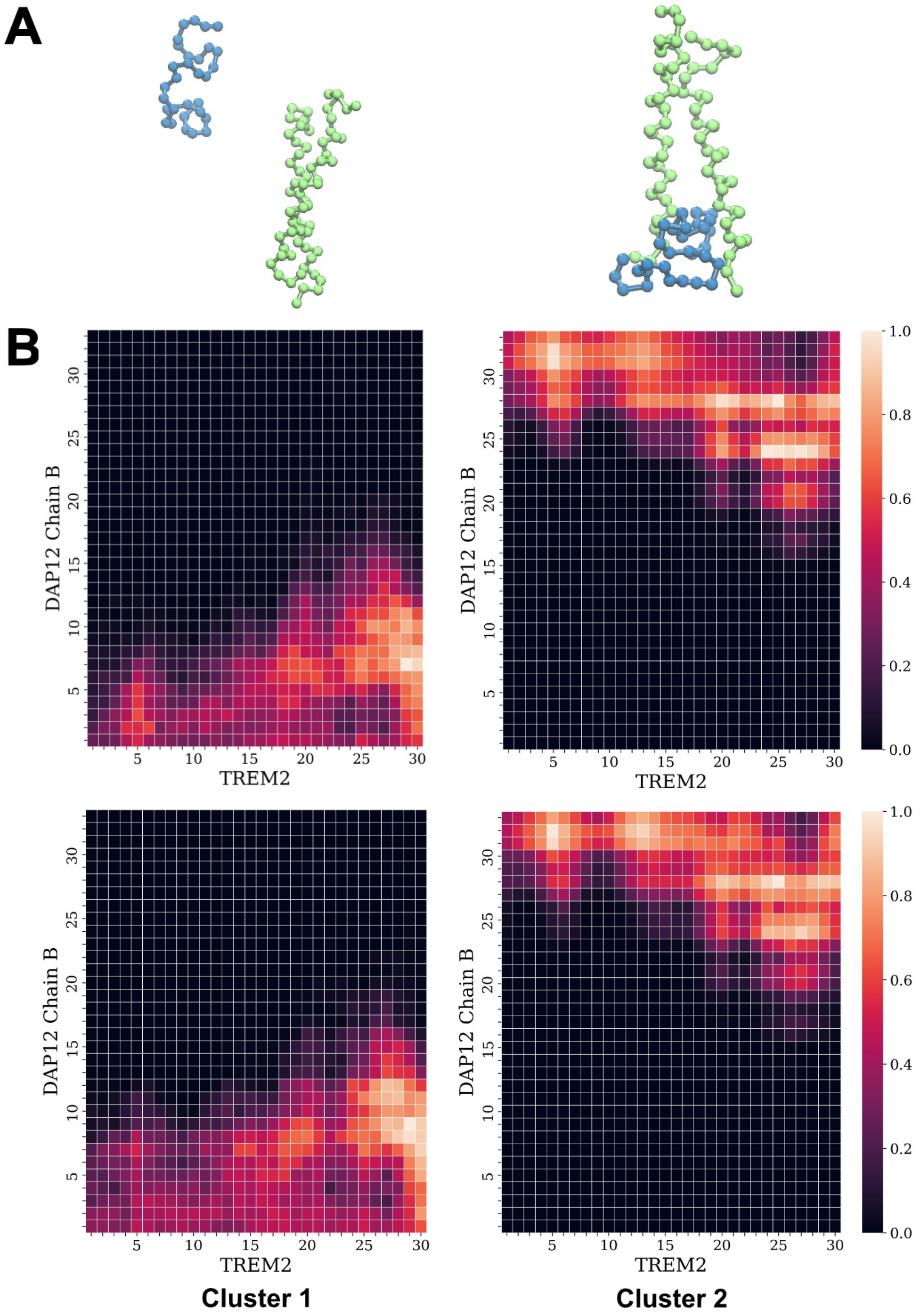

Figure S8: Structural ensembles and contact frequency maps of the W191X-219 system. (A) Representative transmembrane conformations of Cluster 1 and Cluster 2 derived from MD trajectory clustering. (B) Corresponding contact frequency maps between TREM2 and DAP12 Chain A and Chain B for each cluster. Color scale denotes contact frequency from low (black) to high (white). Both Cluster 1 and Cluster 2 display dispersed contact patterns, suggesting a destabilized interface and a shift in binding interactions upon truncation. Notably, Cluster 1 shows contacts between TREM2 and the N-terminal region of DAP12, while Cluster 2 exhibits contacts primarily in the upper transmembrane region.

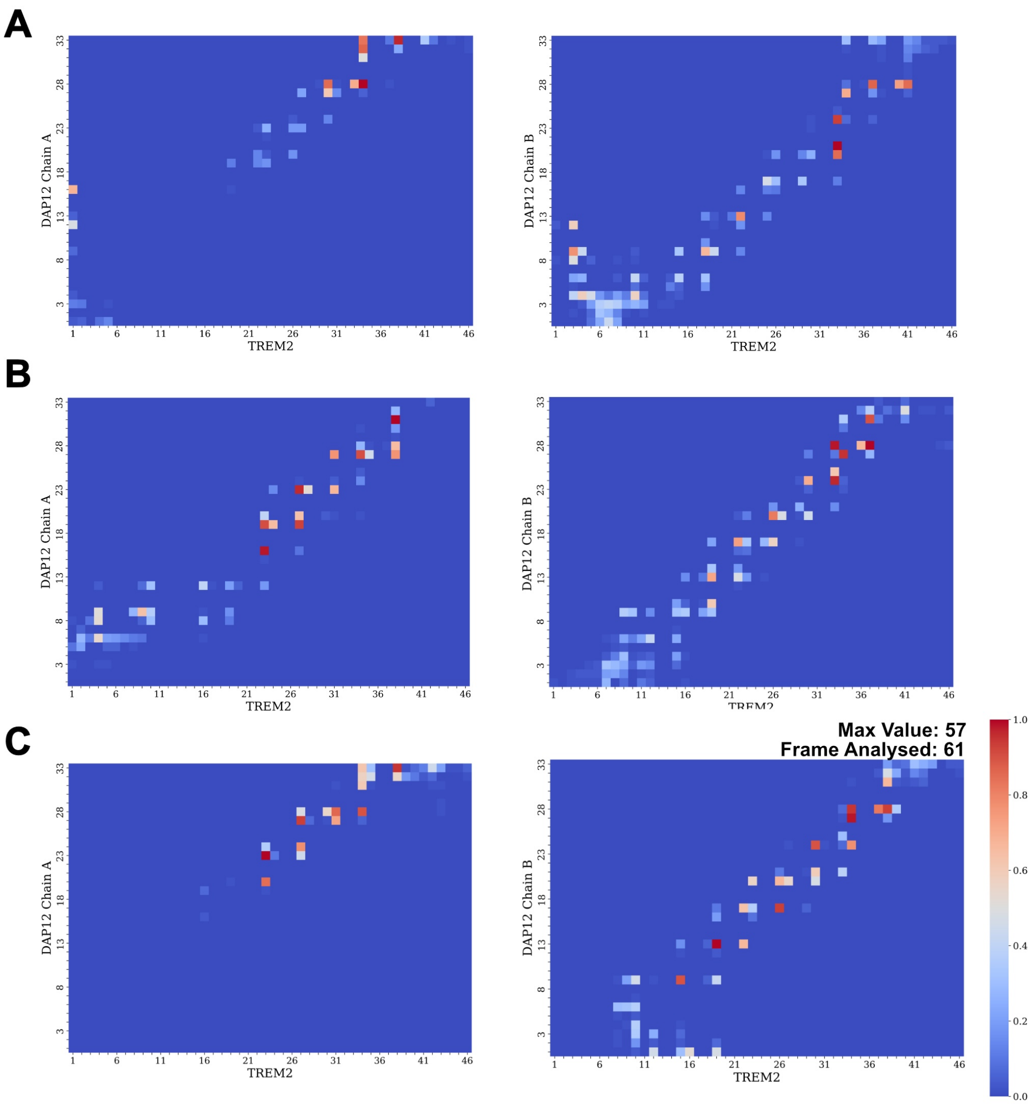

Figure S9: Contact frequency maps of DAP12 with TREM2 in different clusters of the K186A-230 system. (A-C) Contact frequency maps for Cluster 4 (290 μs), Cluster 1 (295 μs), and Cluster 3 (298 μs), respectively. Each panel shows contacts between TREM2 and DAP12 Chain A (left) and Chain B (right). Color scale indicates contact frequency from low (blue) to high (red), normalized to a maximum value of 1.0. The similarity across panels A-C highlights consistent interaction patterns in replicate trajectories of the K186A-230 system.

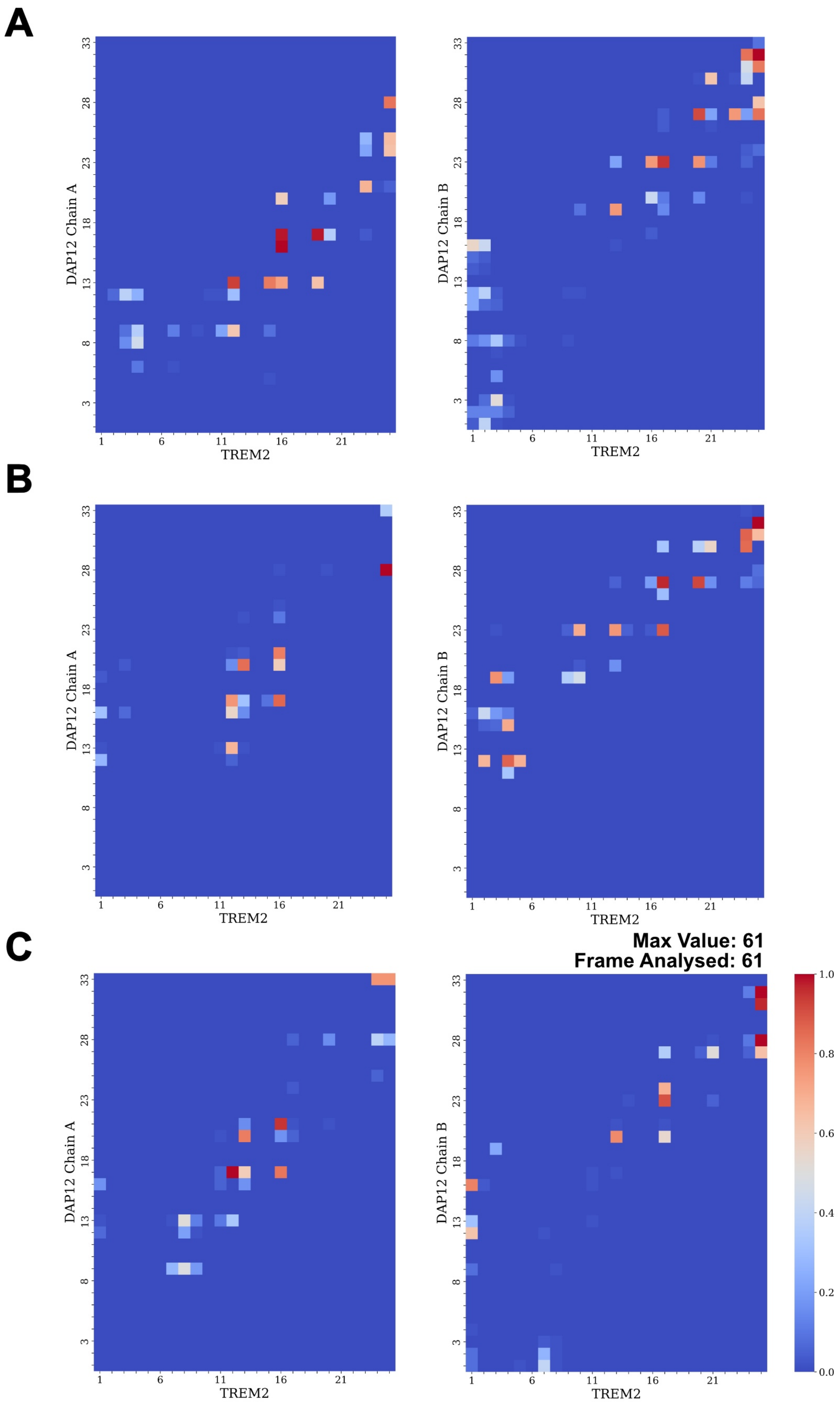

Figure S10: Contact frequency maps of DAP12 with TREM2 in different clusters of the K186X-230 system. (A-C) Contact frequency maps for Cluster 2 (297 μs), Cluster 4 (299 μs), and Cluster 4 (300 μs), respectively. Left and right panels in each subfigure show contact frequencies between TREM2 and DAP12 Chain A and Chain B. The color scale indicates contact frequency, ranging from low (blue) to high (red), normalized to a maximum value of 1.0.

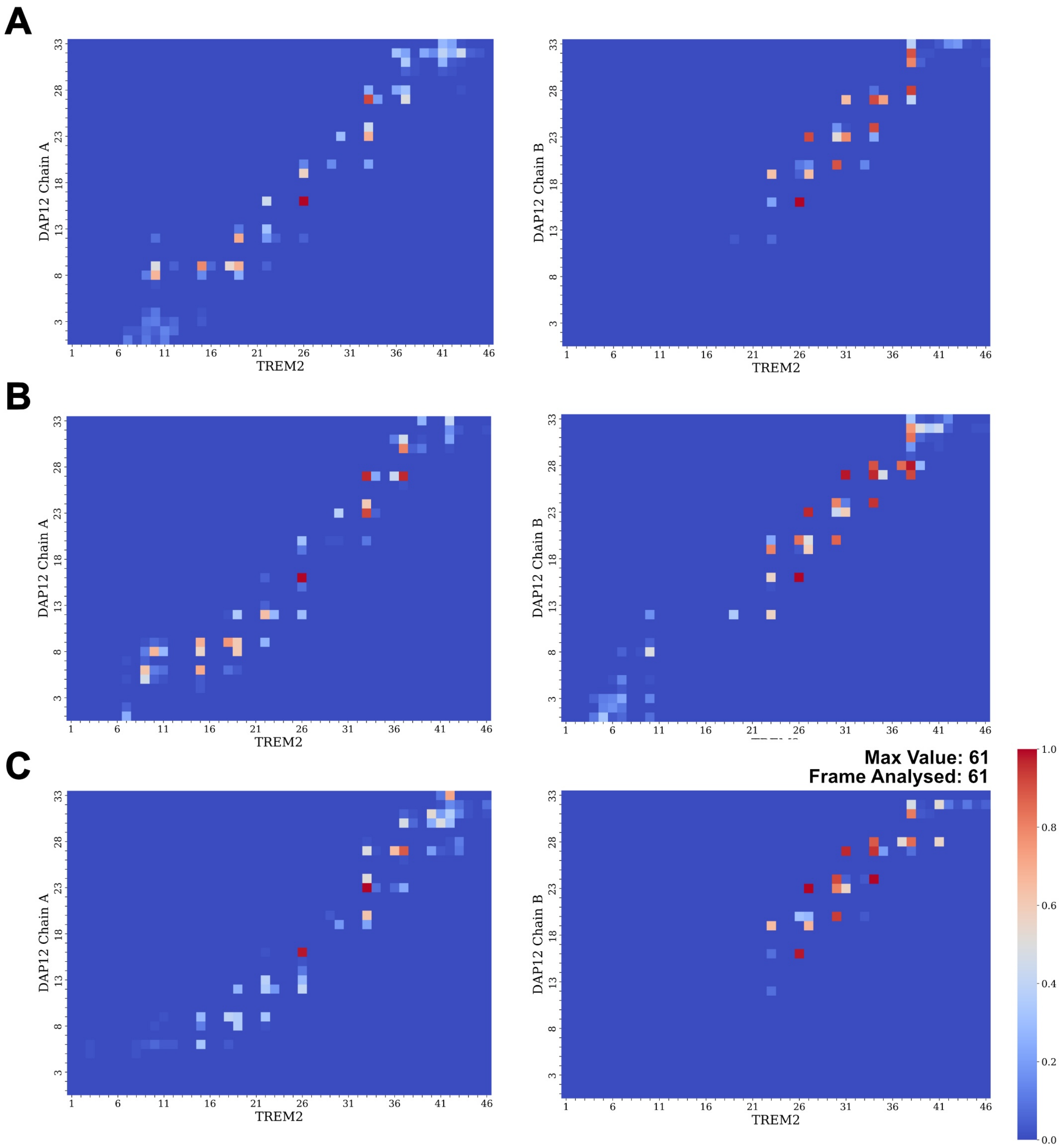

Figure S11: Contact frequency maps of DAP12 with TREM2 in replicate trajectories of the W194A-230 system. (A-C) Contact frequency maps for Cluster 4 at 297 μs, 299 μs, and 300 μs, respectively. Left and right panels in each subfigure show contact frequencies between TREM2 and DAP12 Chain A and Chain B. The color scale represents contact frequency from low (blue) to high (red), normalized to a maximum of 1.0. Consistent patterns across panels A-C indicate reproducible interaction profiles in the W194A-230 system.

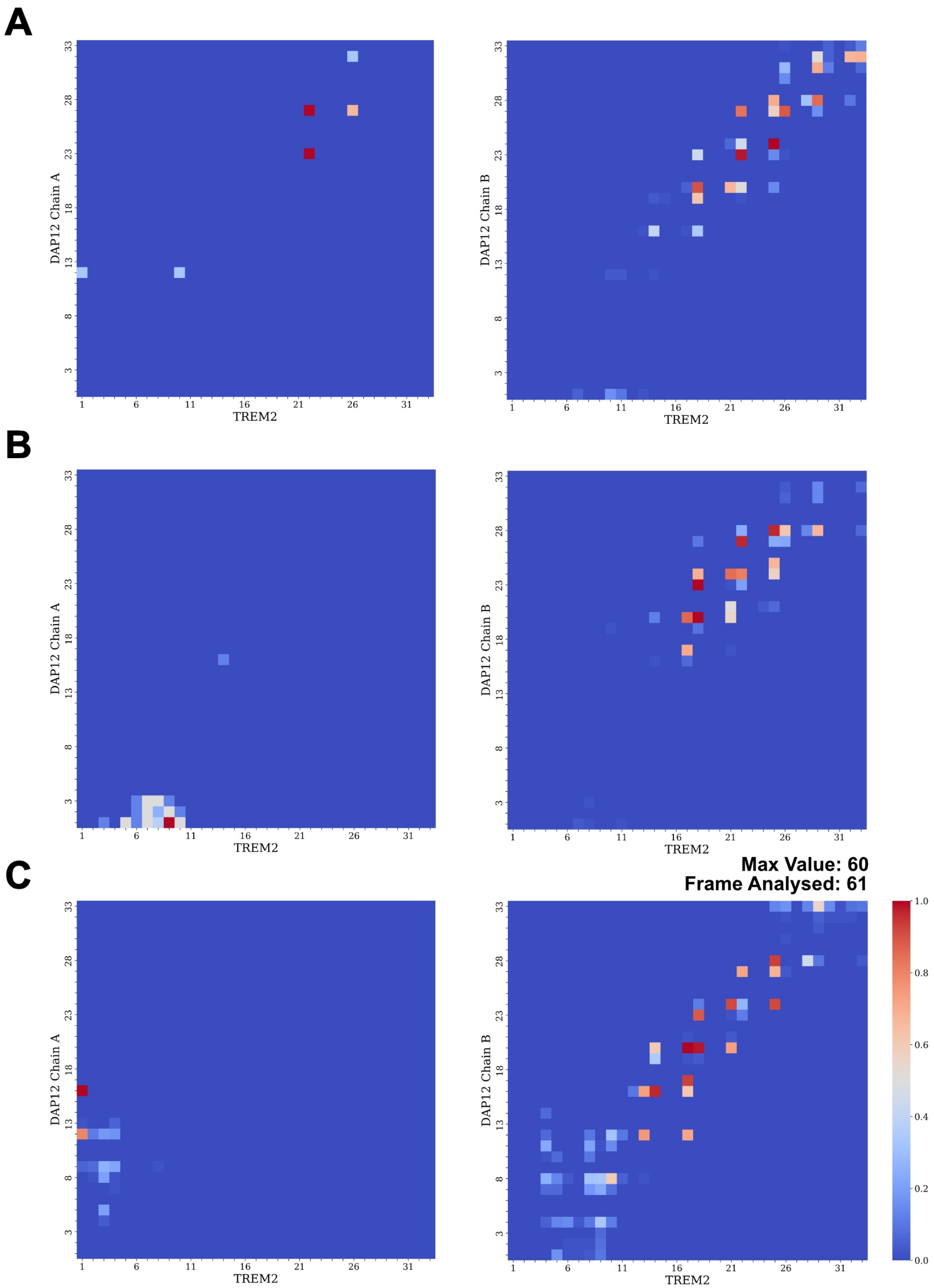

Figure S12: Contact frequency maps of DAP12 with TREM2 in replicate trajectories of the W194X-230 system. (A-C) Contact frequency maps for Cluster 2 at 297 μs, 299 μs, and 300 μs, respectively. Left and right panels in each subfigure show contact frequencies between TREM2 and DAP12 Chain A and Chain B. The color scale represents contact frequency from low (blue) to high (red), normalized to a maximum of 1.0.

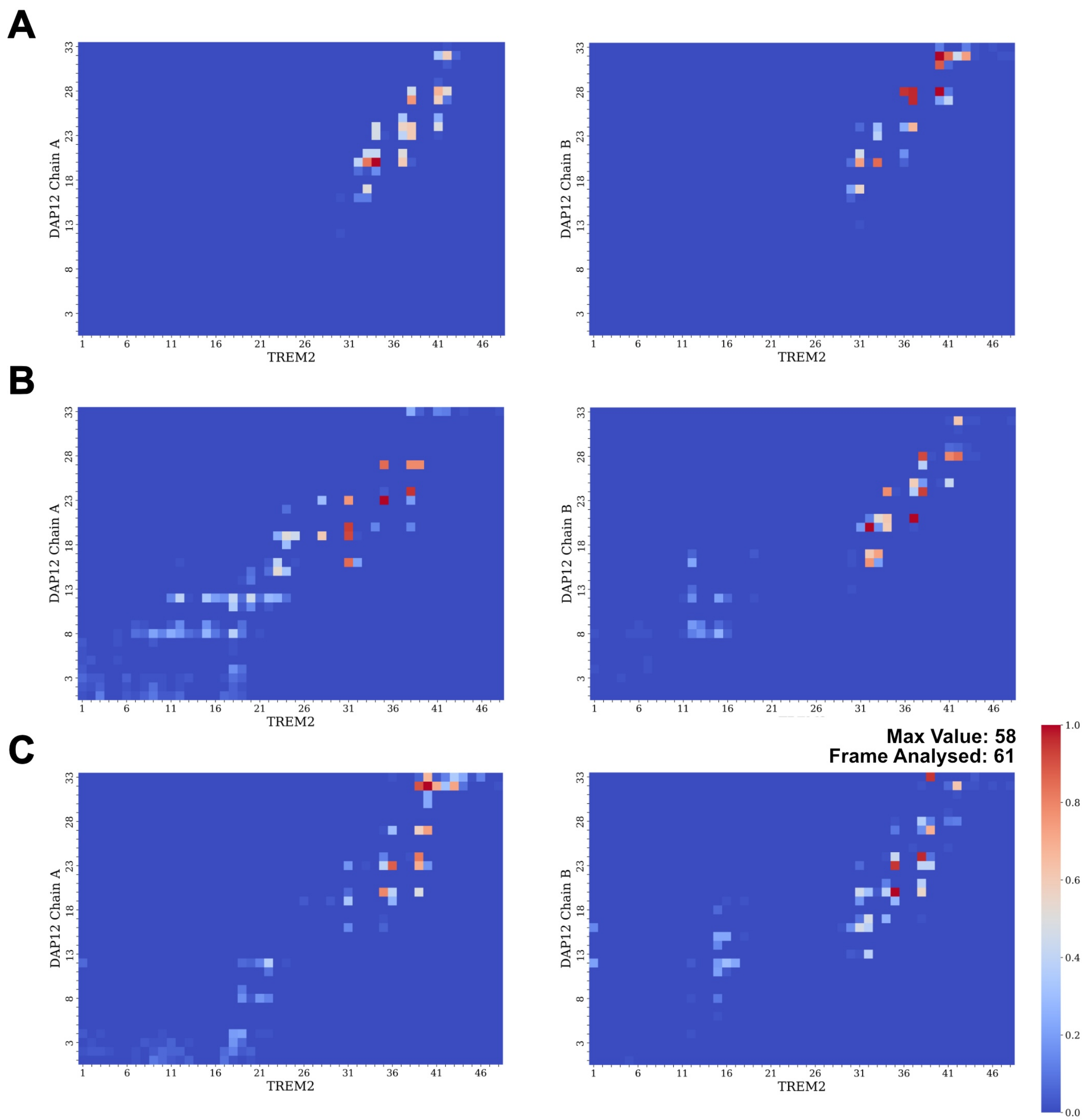

Figure S13: Contact frequency maps of DAP12 with TREM2 in different clusters of the WT-219 system. (A-C) Contact frequency maps for Cluster 1 at 198 μs, and Cluster 2 at 199 μs and 200 μs, respectively. Left and right panels in each subfigure represent contact frequencies between TREM2 and DAP12 Chain A and Chain B. Color scale represents contact frequency from low (blue) to high (red), normalized to a maximum of 1.0.

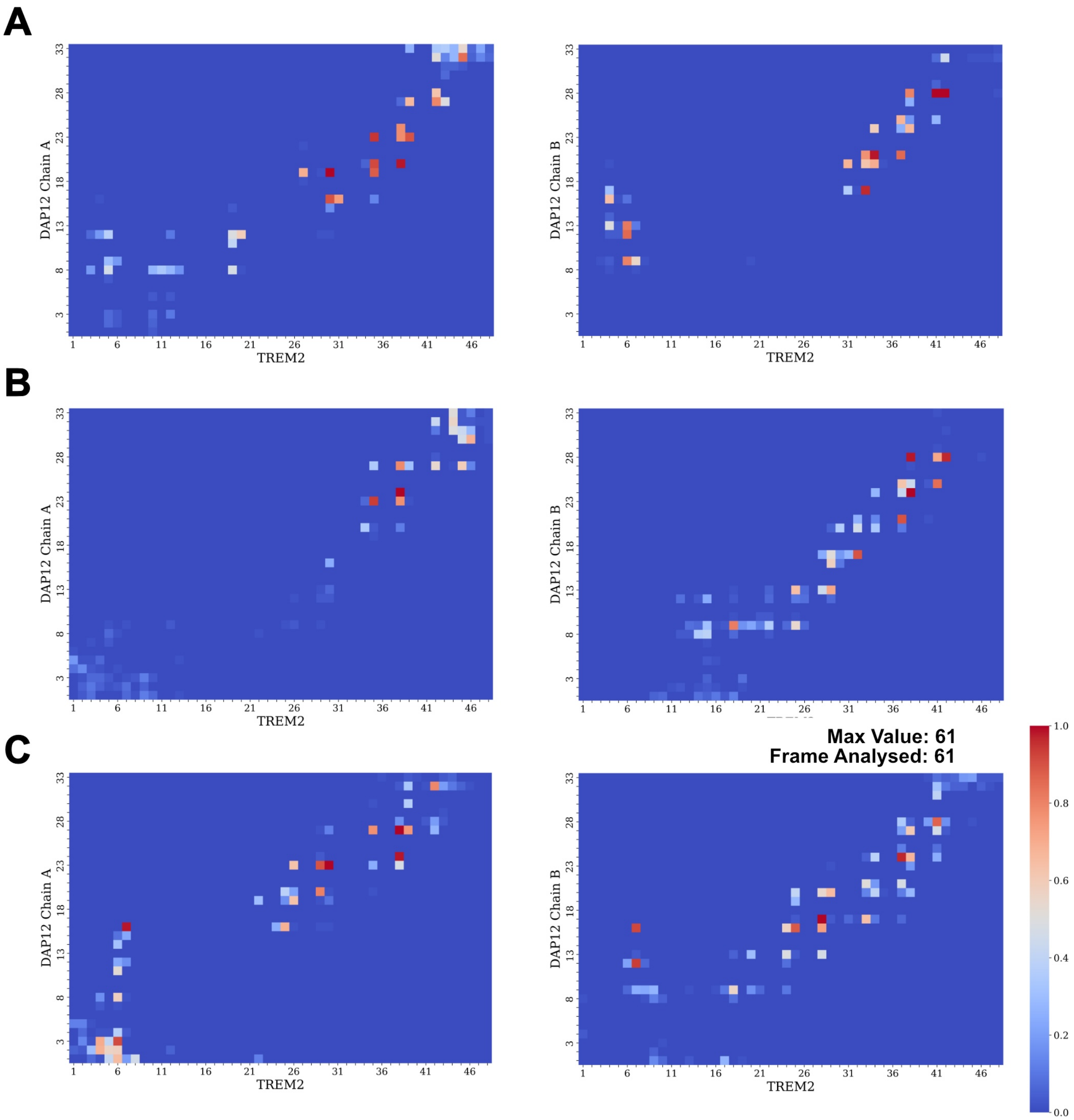

Figure S14: Contact frequency maps of DAP12 with TREM2 in different clusters of the W191A-219 system. (A-C) Contact frequency maps for Cluster 2 at 198 μs, and Cluster 1 at 199 μs and 200 μs, respectively. Left and right panels in each subfigure show contact frequencies between TREM2 and DAP12 Chain A and Chain B. The color scale represents contact frequency from low (blue) to high (red), normalized to a maximum of 1.0.

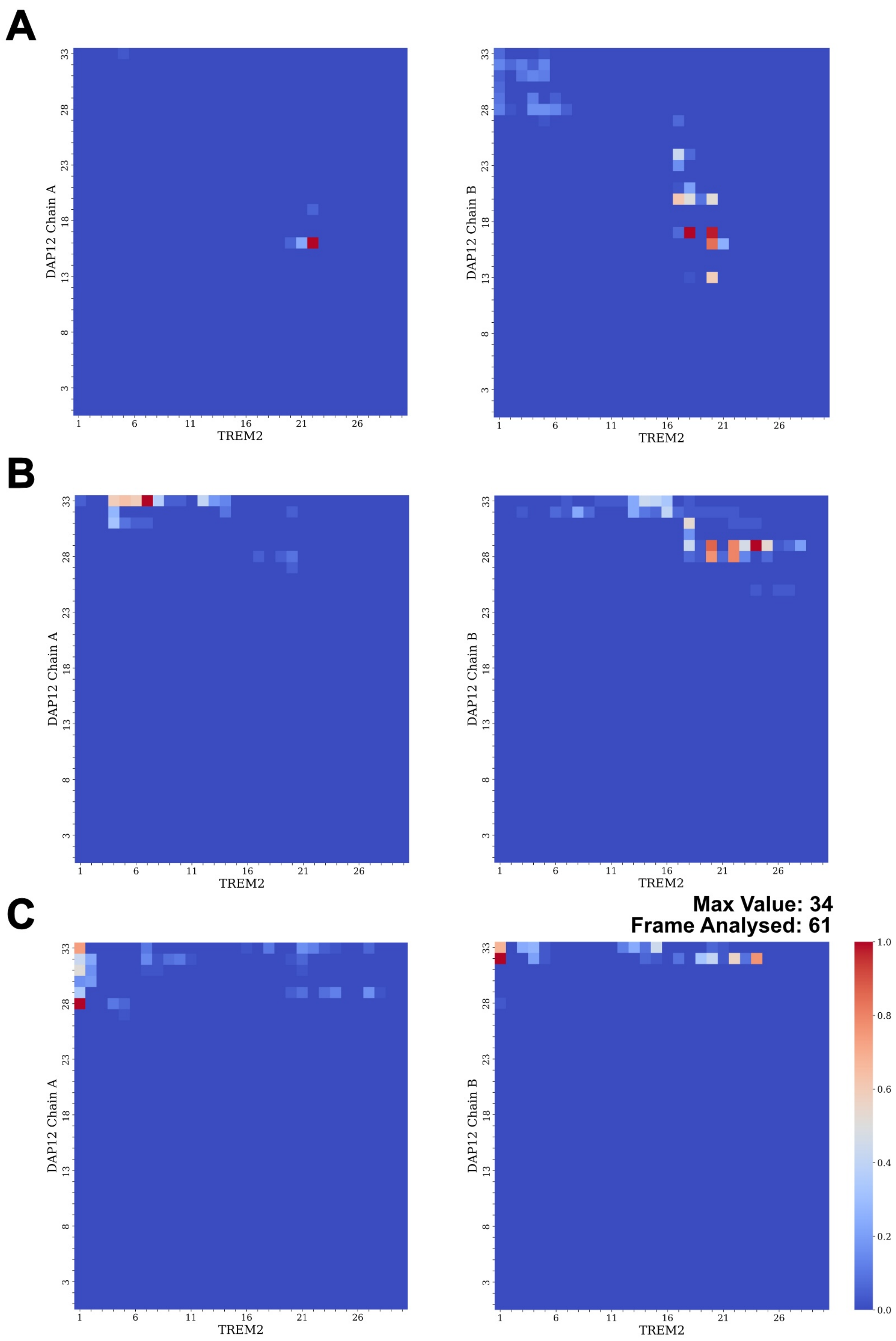

Figure S15: Contact frequency maps of DAP12 with TREM2 in different replicates of the W191X-219 system, all corresponding to Cluster 2. (A-C) Contact frequency maps for simulations at 197 μs, 198 μs, and 200 μs, respectively. Left and right panels in each subfigure display contact frequencies between TREM2 and DAP12 Chain A and Chain B. The color scale represents contact frequency from low (blue) to high (red), normalized to a maximum of 1.0.

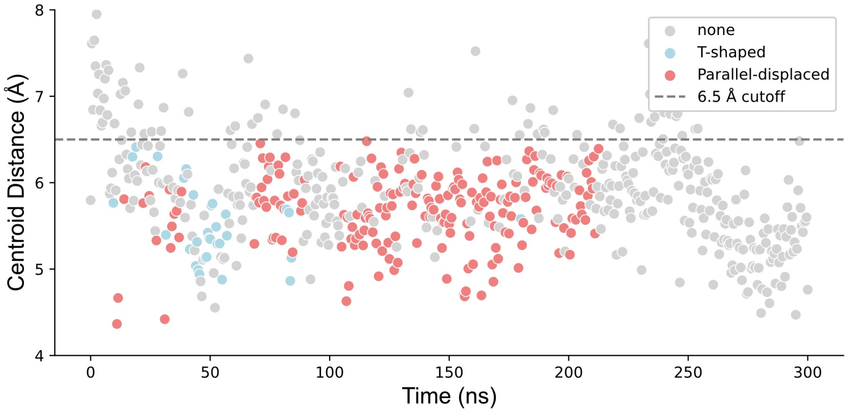

Figure S16: **Hydrogen bonding between TREM2 K186 and DAP12 D50 is maintained in the W194A-230 mutant.** The number of hydrogen bonds (N_{HB}) formed between the side chain of K186 (TREM2) and D50 (DAP12) over time in the W194A-230 mutant. A stable hydrogen bond network is observed throughout the 300 ns simulation: two hydrogen bonds occur in 53.2% of frames, and one bond in 39.1% of frames, with bonding present in 92.3% of the total trajectory. These data support the persistence of this key interfacial interaction in stabilizing the TREM2-DAP12 complex in the W194A-230 mutant.

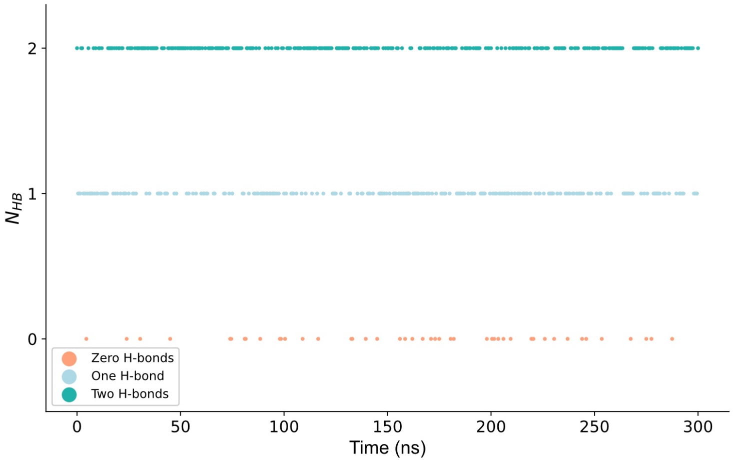

Figure S17: **Time-resolved quantification of hydrogen bonds between K186 of TREM2 and D50 of DAP12 in the W194A-230 mutant system.** Each point represents the number of hydrogen bonds (N_{HB}) formed at a specific time frame across the 300 ns simulation. Hydrogen bonding is categorized into three states: zero (orange), one (light blue), and two (teal) hydrogen bonds. This persistent bonding supports the formation of a tightly packed trimer interface.

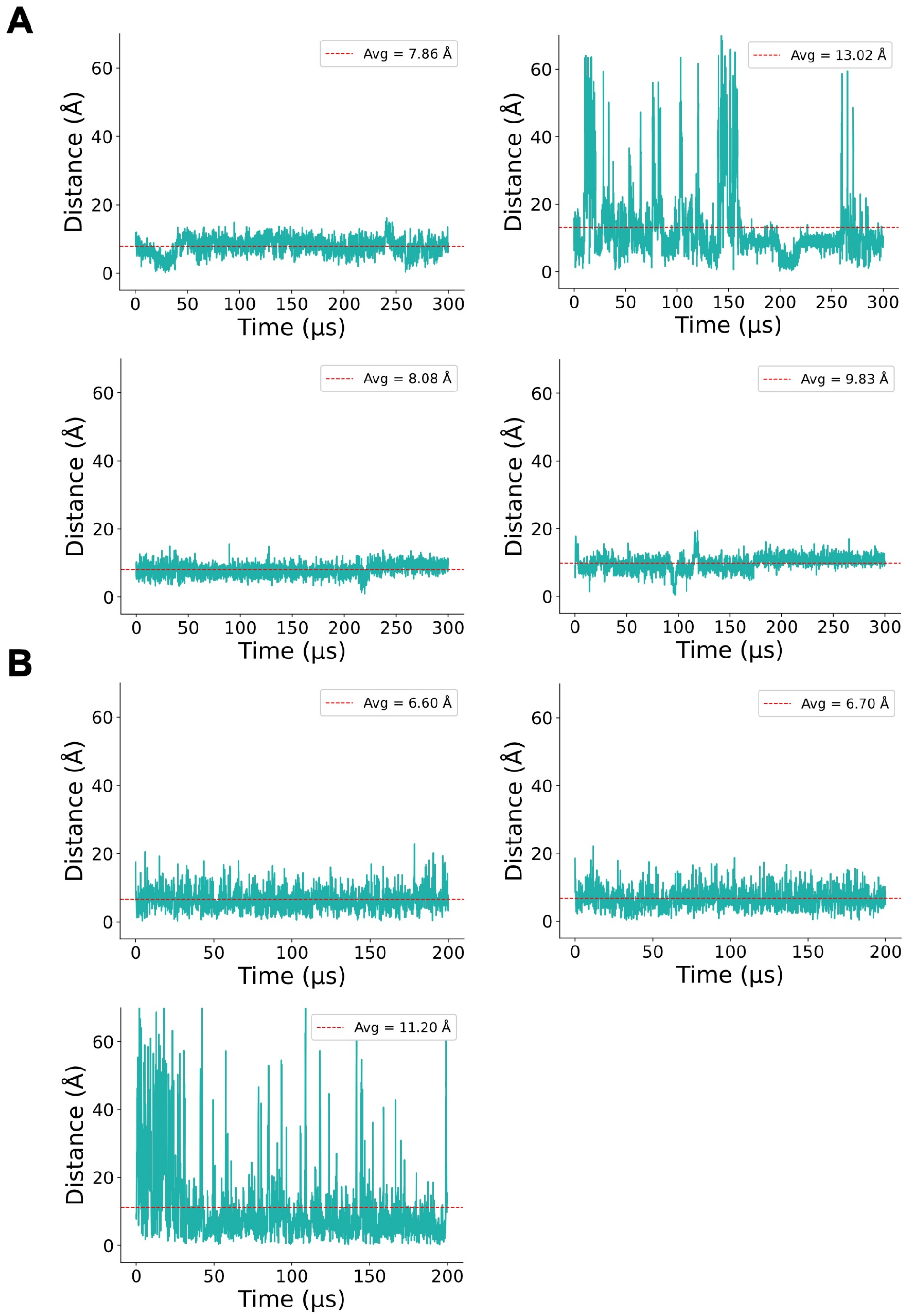

Figure S18: **Centre-of-mass (COM) distance analysis reveals destabilised interfaces in specific TREM2-DAP12 mutants. (A)** COM distances between TREM2 and DAP12 in the 230-residue isoform for K186A, K186X, W191A, and W191X mutants. The K186X-230 mutant shows a markedly higher average distance (13.02 Å), suggesting a disrupted or weakened interface, whereas K186A-230, W194A-230, and W194X-230 maintain shorter average distances (7.86 Å, 8.08 Å, and 9.83 Å, respectively). **(B)** COM distances for the 219-residue isoform in WT, W191A, and W191X constructs. WT-219 and W191A-219 maintain tight packing (6.60 Å and 6.70 Å), while W191X-219 shows significantly increased spatial separation (11.20 Å).

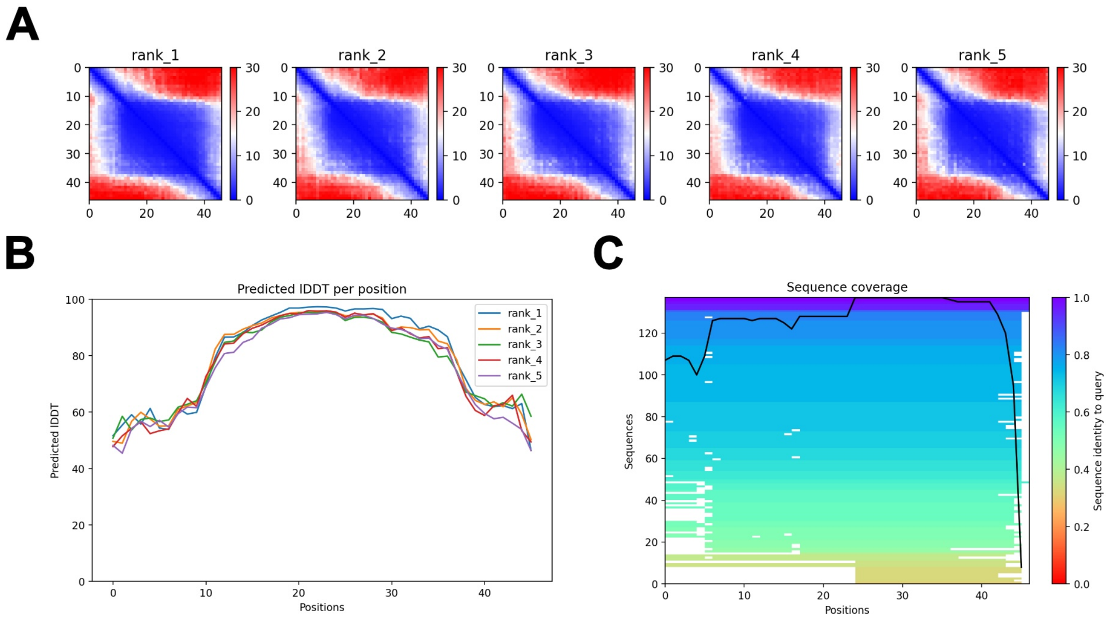

Figure S19: **ColabFold prediction confidence for the TREM2 K186A-230 mutant. (A)** Predicted aligned error (PAE) plots for five AlphaFold2 models (rank_1 to rank_5). **(B)** Predicted lDDT scores per residue for each model. **(C)** Multiple sequence alignment (MSA) coverage and identity plotted across the K186A sequence. The black trace indicates overall sequence coverage.

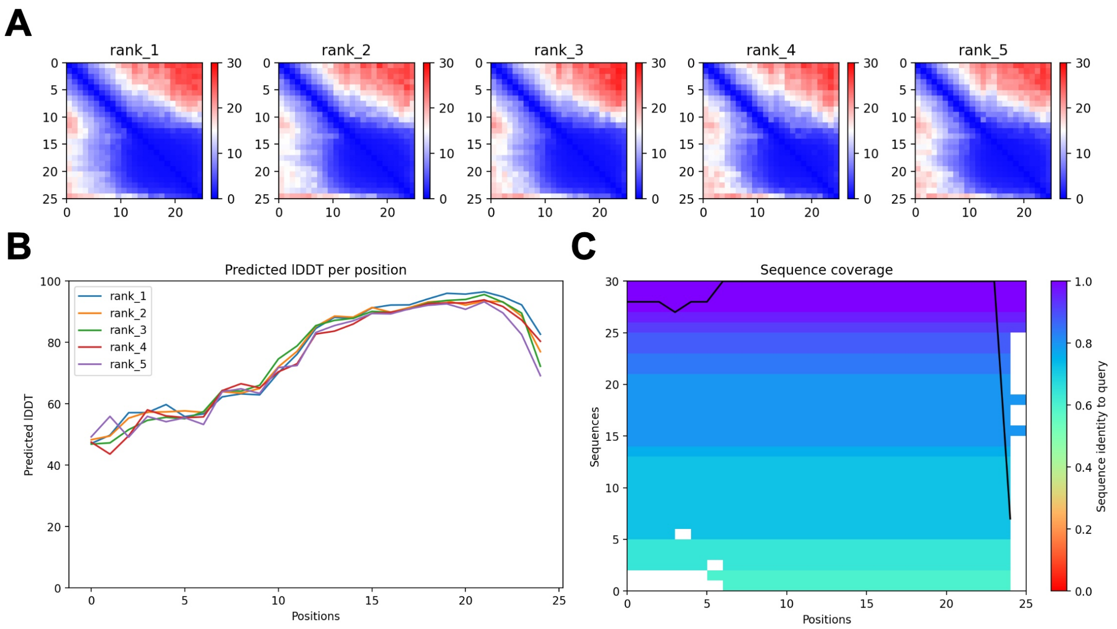

Figure S20: **ColabFold prediction confidence for the TREM2 K186X-230 mutant. (A)** Predicted aligned error (PAE) plots for five AlphaFold2 models (rank_1 to rank_5). **(B)** Predicted IDDT scores per residue for each model. **(C)** Multiple sequence alignment (MSA) coverage and identity plotted across the K186X sequence. The black trace indicates overall sequence coverage.

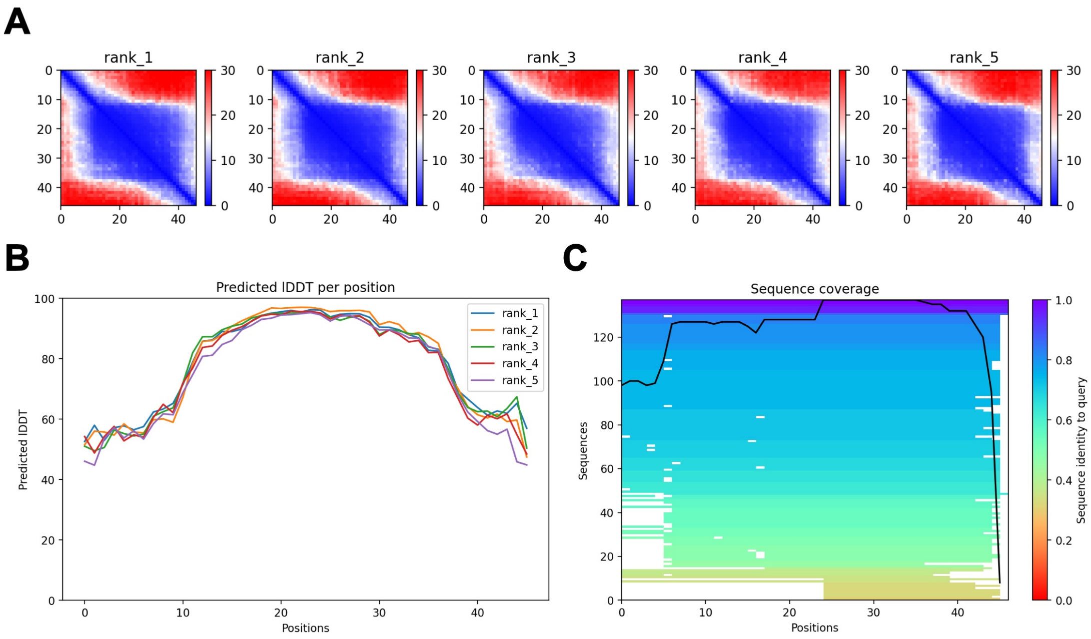

Figure S21: **ColabFold prediction confidence for the TREM2 W194A-230 mutant.** **(A)** Predicted aligned error (PAE) plots for five AlphaFold2 models (rank_1 to rank_5). **(B)** Predicted IDDT scores per residue for each model. **(C)** Multiple sequence alignment (MSA) coverage and identity plotted across the K186X sequence. The black trace indicates overall sequence coverage.

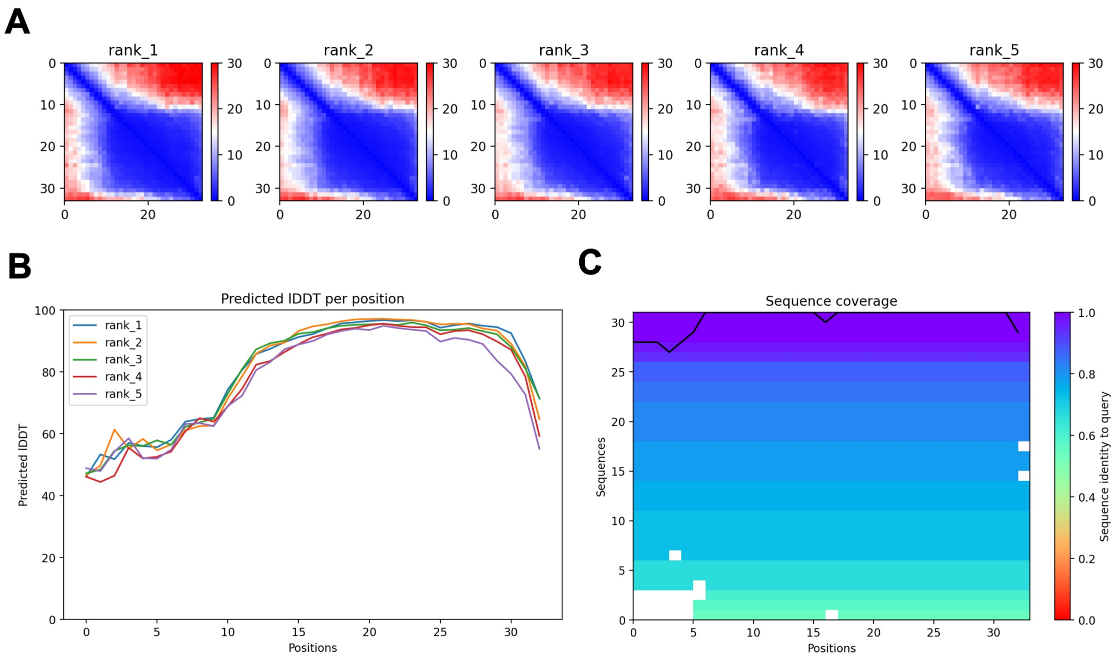

Figure S22: **ColabFold prediction confidence for the TREM2 W194X-230 mutant.** **(A)** Predicted aligned error (PAE) plots for five AlphaFold2 models (rank_1 to rank_5). **(B)** Predicted IDDT scores per residue for each model. **(C)** Multiple sequence alignment (MSA) coverage and identity plotted across the K186X sequence. The black trace indicates overall sequence coverage.

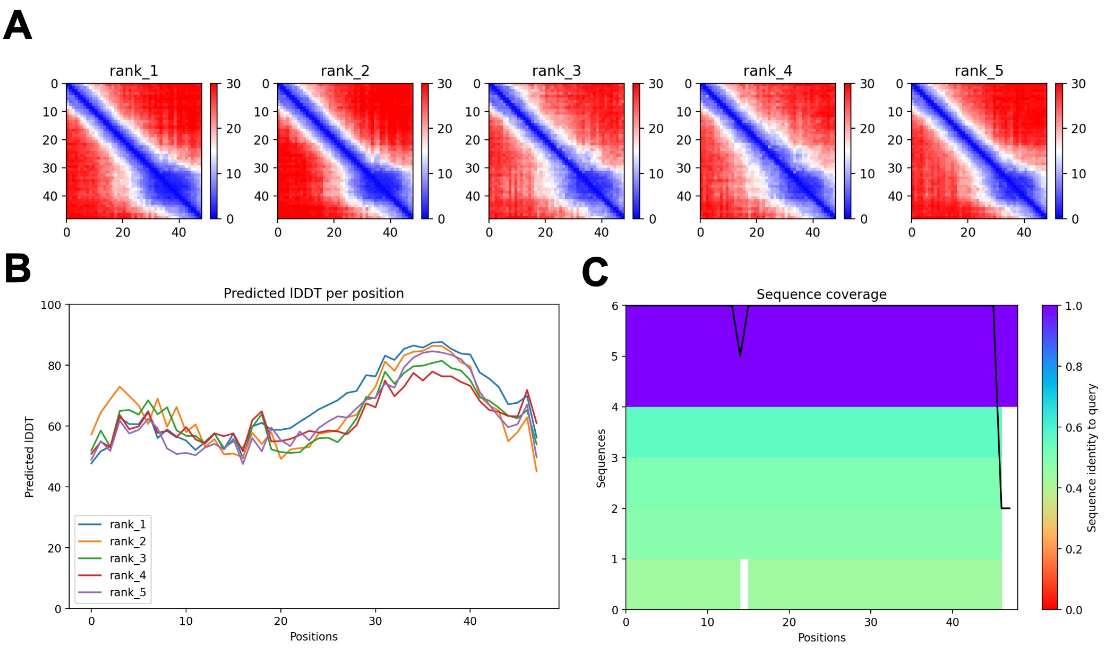

Figure S23: **ColabFold prediction confidence for the TREM2 WT-219 mutant.** **(A)** Predicted aligned error (PAE) plots for five AlphaFold2 models (rank_1 to rank_5). **(B)** Predicted IDDT scores per residue for each model. **(C)** Multiple sequence alignment (MSA) coverage and identity plotted across the K186X sequence. The black trace indicates overall sequence coverage.

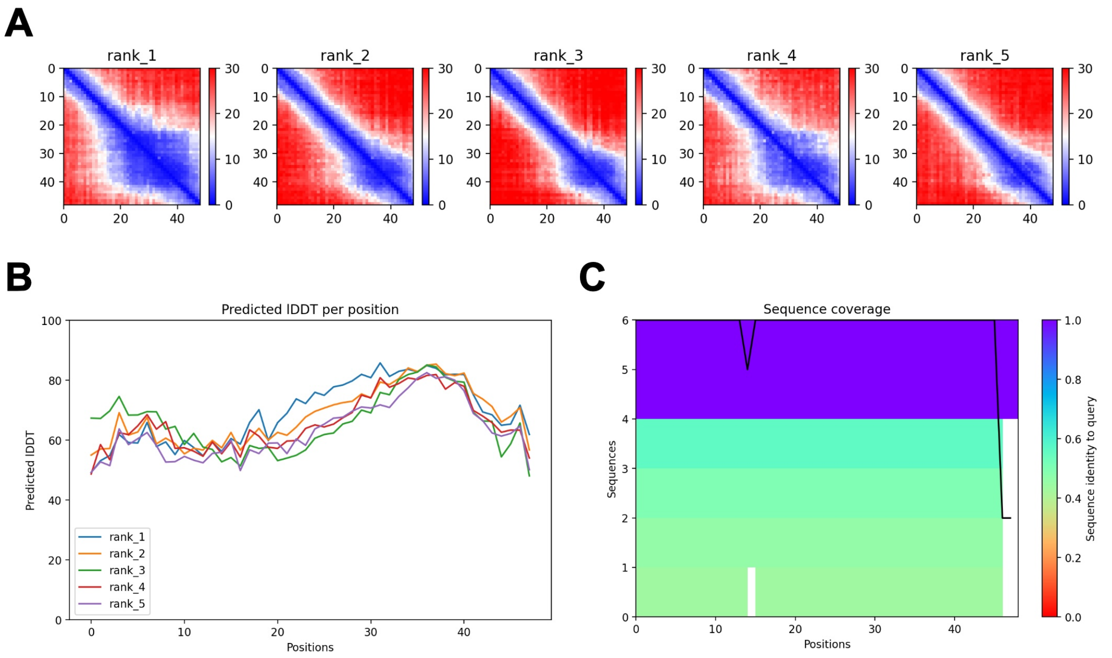

Figure S24: **ColabFold prediction confidence for the TREM2 W191A-219 mutant.** **(A)** Predicted aligned error (PAE) plots for five AlphaFold2 models (rank_1 to rank_5). **(B)** Predicted IDDT scores per residue for each model. **(C)** Multiple sequence alignment (MSA) coverage and identity plotted across the K186X sequence. The black trace indicates overall sequence coverage.

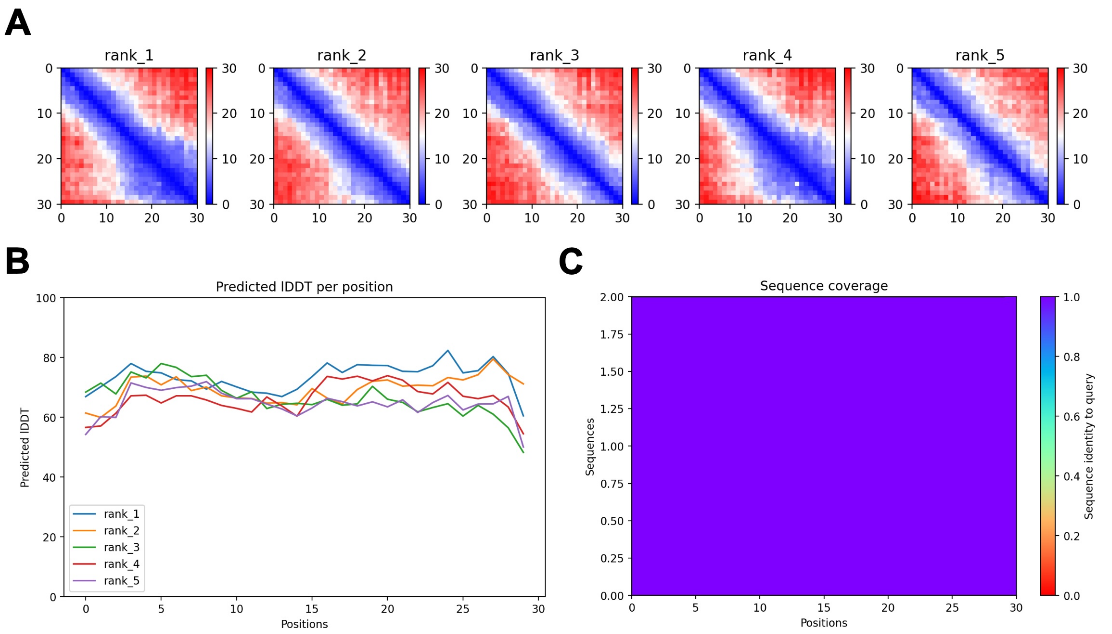

Figure S25: **ColabFold prediction confidence for the TREM2 W191X-219 mutant.** **(A)** Predicted aligned error (PAE) plots for five AlphaFold2 models (rank_1 to rank_5). **(B)** Predicted lDDT scores per residue for each model. **(C)** Multiple sequence alignment (MSA) coverage and identity plotted across the K186X sequence. The black trace indicates overall sequence coverage.

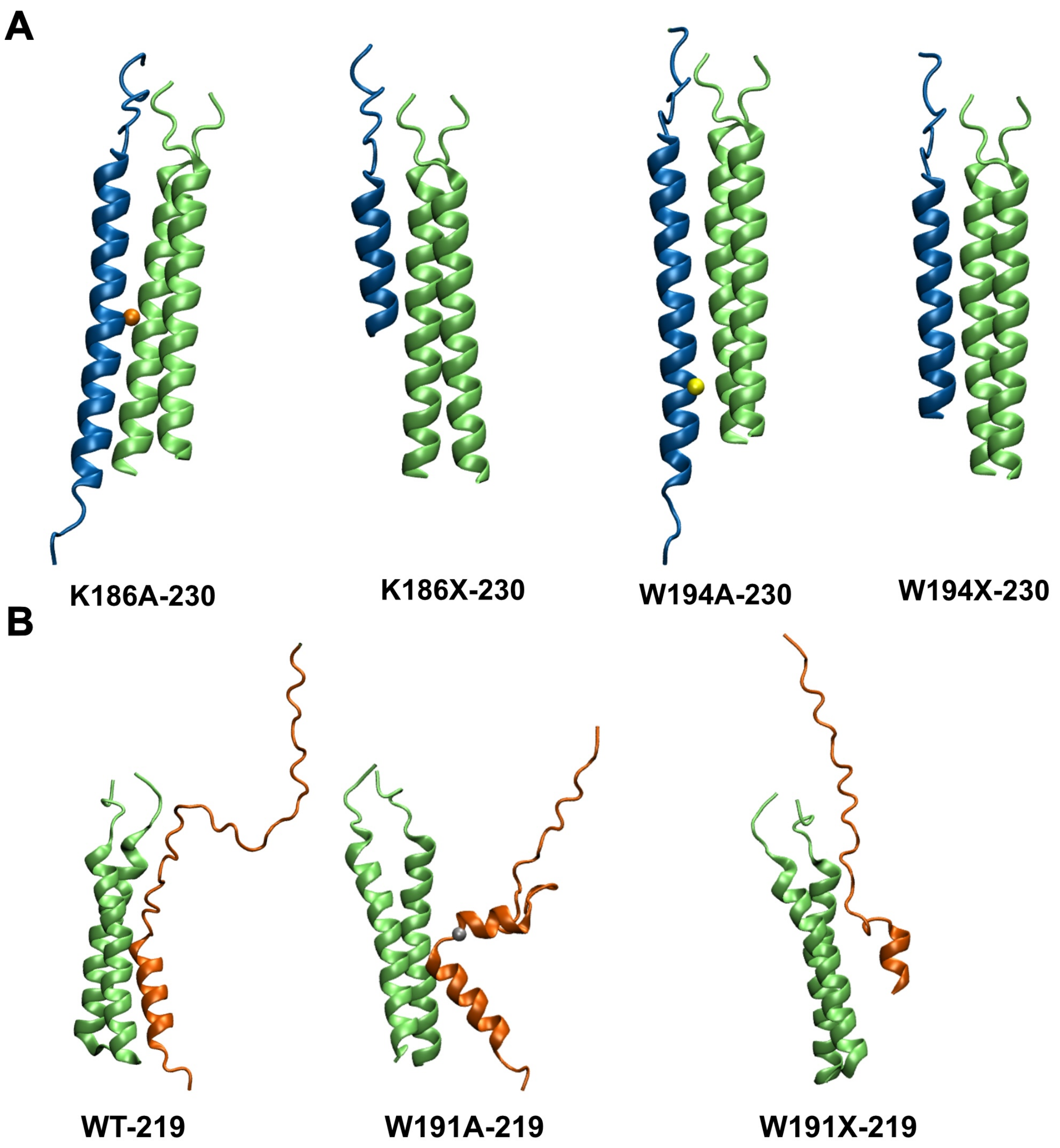

Figure S26: **Initial structures of the TREM2-DAP12 complex for each simulation system.** **(A)** ColabFold-predicted models of TREM2 transmembrane helix variants (residues 186-230 or 194-230) in complex with DAP12. Mutations include K186A, K186X, W194A, and W194X. TREM2 is shown in green, DAP12 in blue, and the mutation sites are highlighted with spheres. **(B)** Initial configurations of wild-type (WT-219), W191A-219, and W191X-219 systems. TREM2 is shown in green, DAP12 in orange, and mutation sites are highlighted.

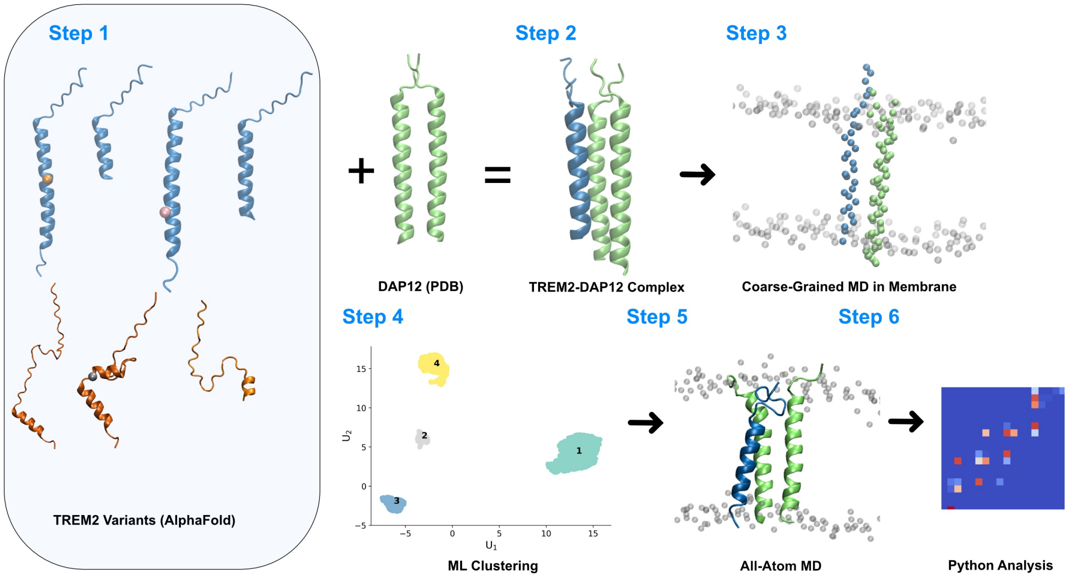

Figure S27: **Computational workflow for investigating TREM2-DAP12 transmembrane complex stability. Step 1:** Structural models of TREM2 isoform 1 and isoform 2 variants were predicted using AlphaFold. **Step 2:** Each TREM2 variant was assembled with DAP12 (PDB: 2L34) to form the initial complex. **Step 3:** The complexes were embedded in a POPC:cholesterol (80:20) bilayer and simulated using coarse-grained (CG) molecular dynamics. **Step 4:** CG trajectories were analyzed with unsupervised machine learning (ML) clustering to identify representative conformational states. **Step 5:** Selected states were converted to atomistic detail for all-atom MD simulations. **Step 6:** Atomistic trajectories were processed using Python-based structural and statistical analyses to quantify mutation effects.

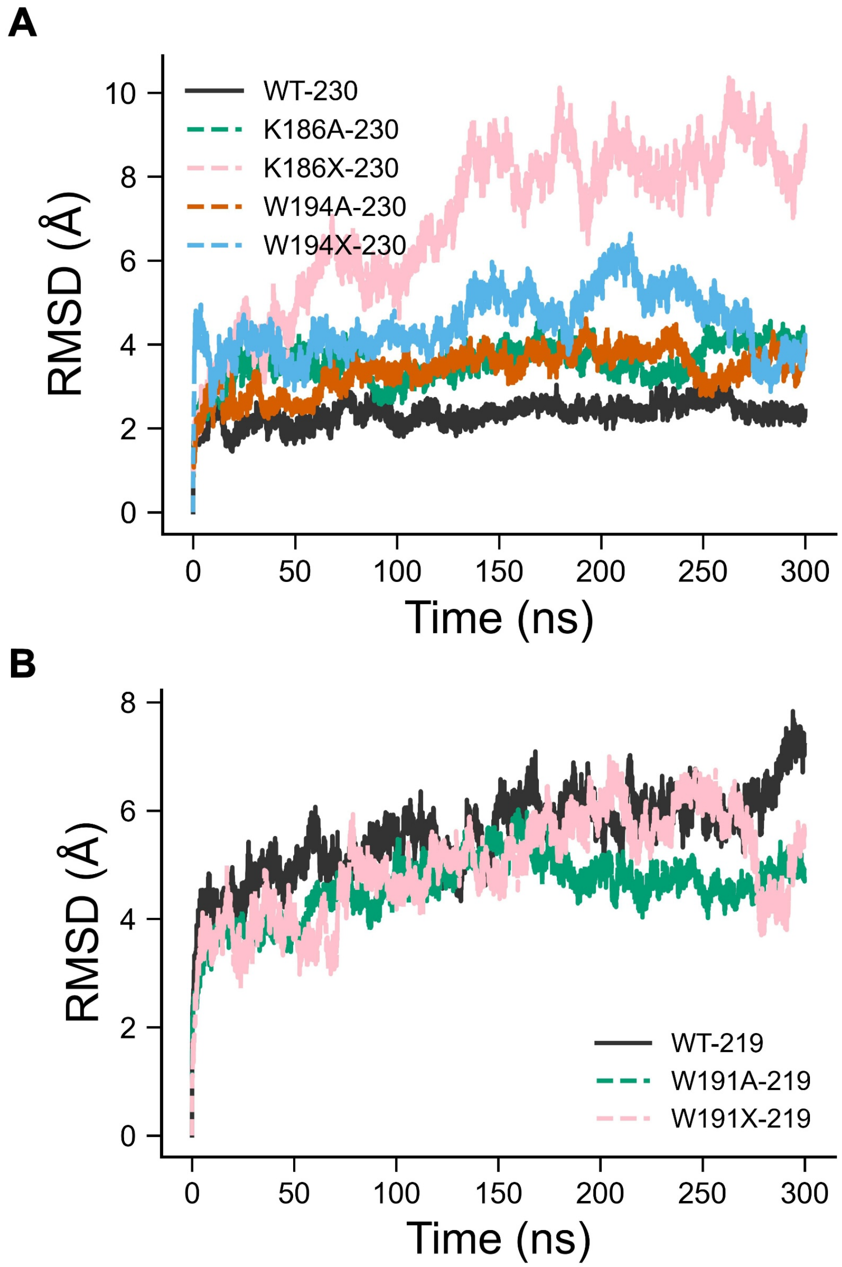

Figure S28: Backbone RMSD analysis of atomistic simulations. (A) RMSD of TREM2-DAP12 isoform-230 systems (WT, K186A/X, and W194A/X variants). (B) RMSD of isoform-219 systems (WT, W191A, and W191X). RMSD values were computed for backbone heavy atoms of DAP12 using the `MDAnalysis.analysis.rms.RMSD` module.

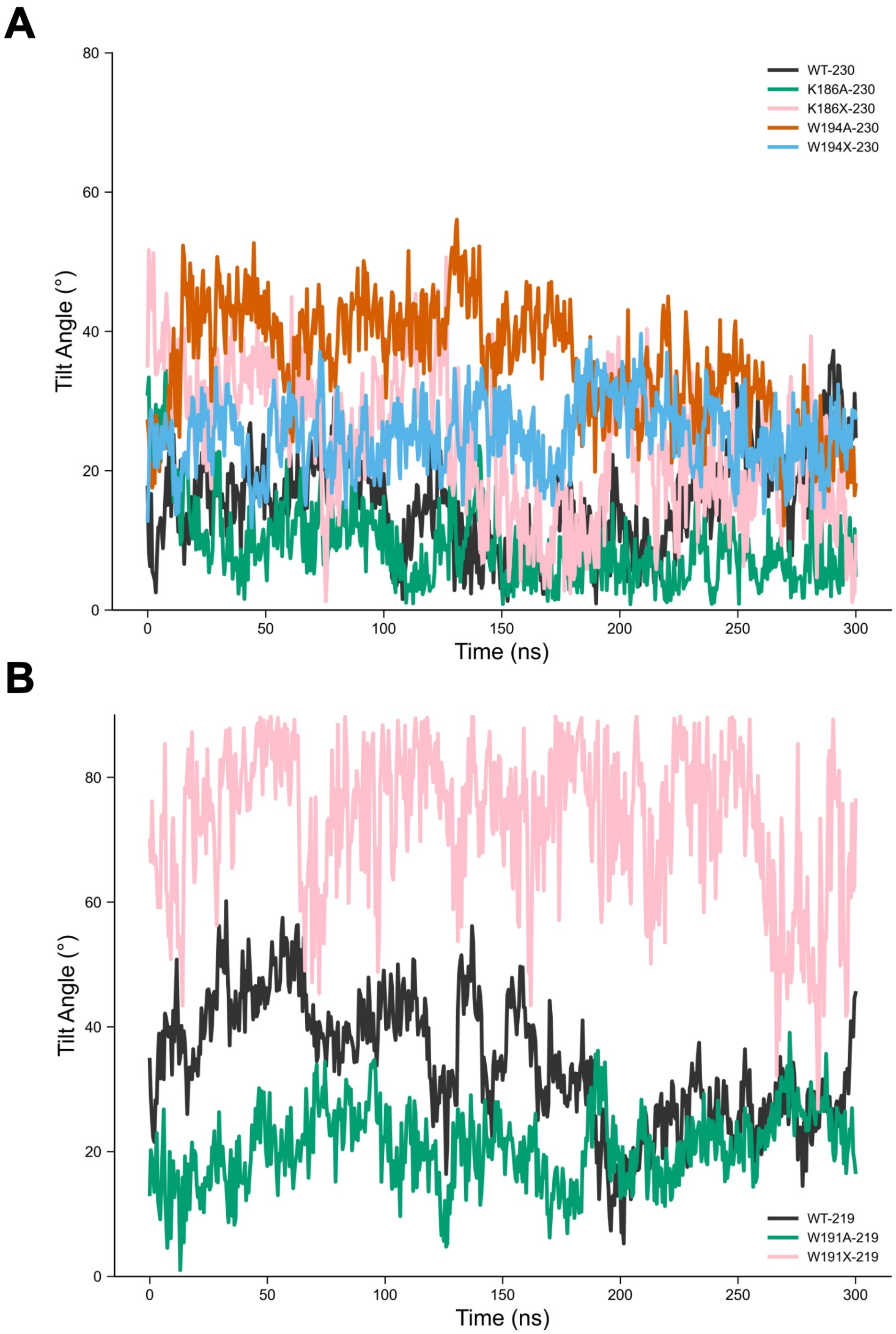

Figure S29: Helix-tilt angle distributions during atomistic simulations. (A) Time evolution of TREM2 transmembrane-helix tilt angles for isoform-230 systems (WT, K186A/X, and W194A/X). (B) Corresponding tilt angles for isoform-219 systems (WT, W191A, and W191X). The tilt angle is defined between the helix-axis vector and the bilayer normal (z-axis). For isoform-230, the helix axis was defined by residues A180 and L184 of TREM2; for isoform-219, by A189 and G194 in the wild type and A185 and A189 in the W191A/X mutants.

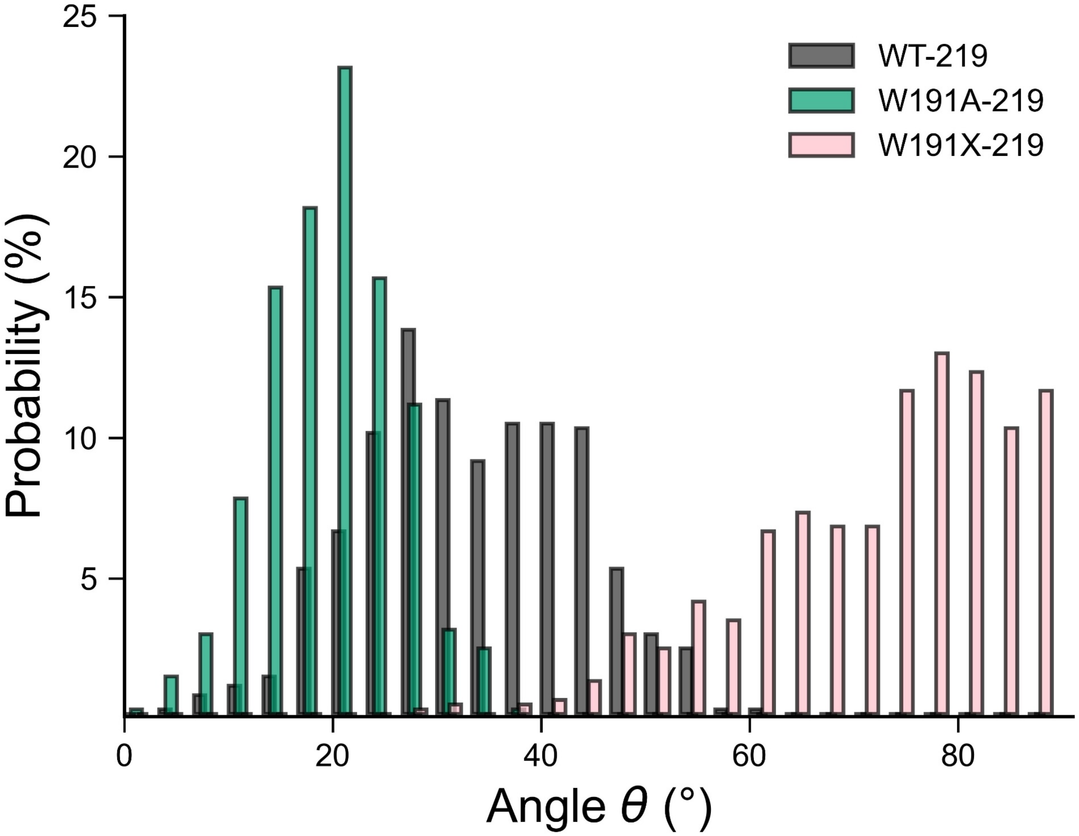

Figure S30: Distribution of TREM2 transmembrane-helix tilt angles during atomistic simulations of isoform-219 systems (WT, W191A, and W191X). Tilt angles were measured between the helix-axis vector, defined by the averaged Cα atoms of residues A189 and G194 in the wild type and A185 and A189 in the W191A/X mutants, and the bilayer normal (z-axis). Histograms represent normalized probability distributions of tilt angles sampled over 300 ns trajectories, showing that W191A maintains a more upright helix orientation, whereas W191X exhibits a markedly higher tilt relative to the WT.

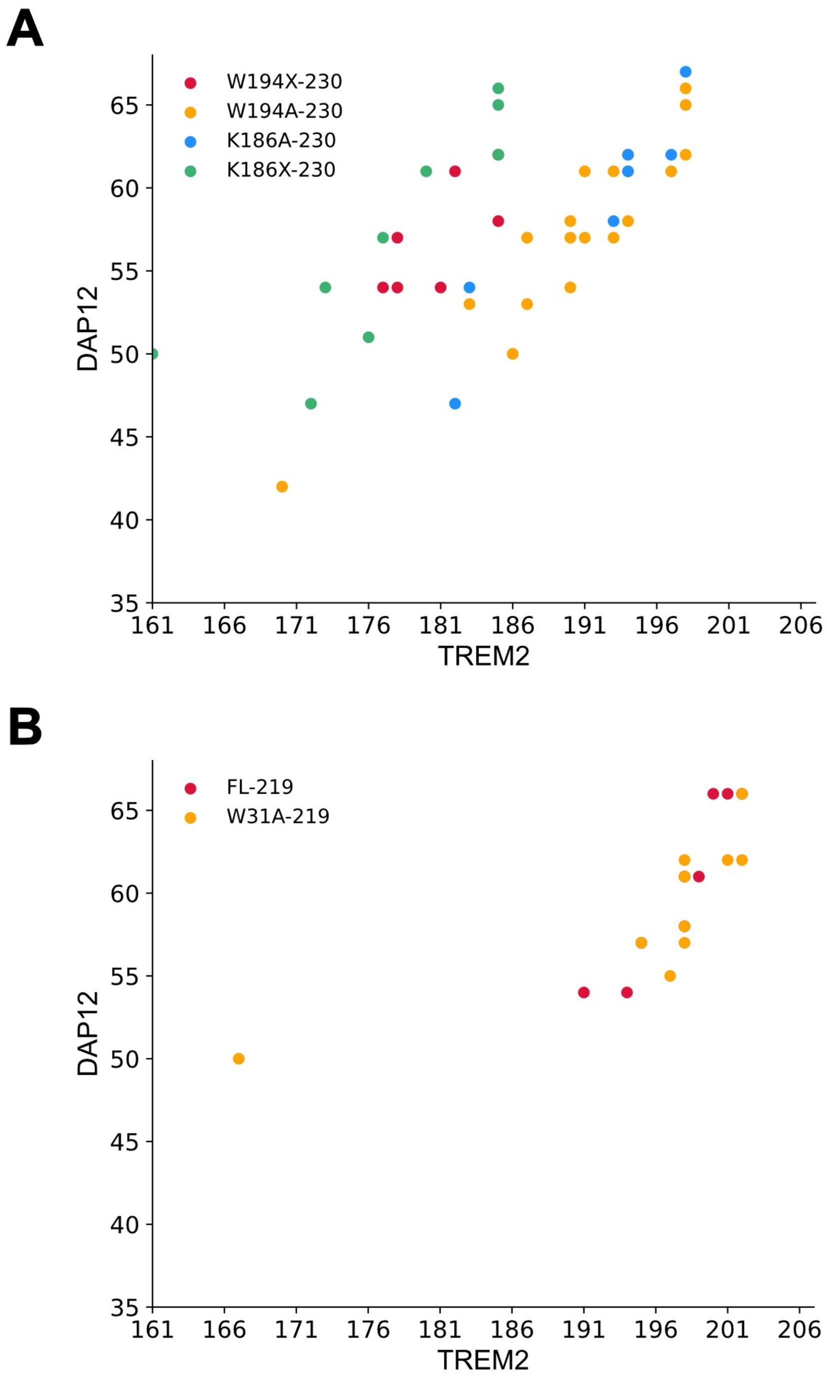

Figure S31: Overlay of key TREM2–DAP12 residue–residue contacts for isoform-230 and isoform-219 systems. (A) Isoform-230 overlays showing contact pairs from WT and mutant complexes (K186A, K186X, W194A, W194X). (B) Isoform-219 overlays showing contacts from WT and mutant systems (W191A and W191X). Each point represents a residue pair within 3.0 Å contact distance averaged over the equilibrated all-atom trajectory. Distinct colors denote different variants, allowing direct visual comparison of contact positions across mutants. The plots highlight mutation-dependent shifts in interfacial contact patterns between TREM2 and DAP12.

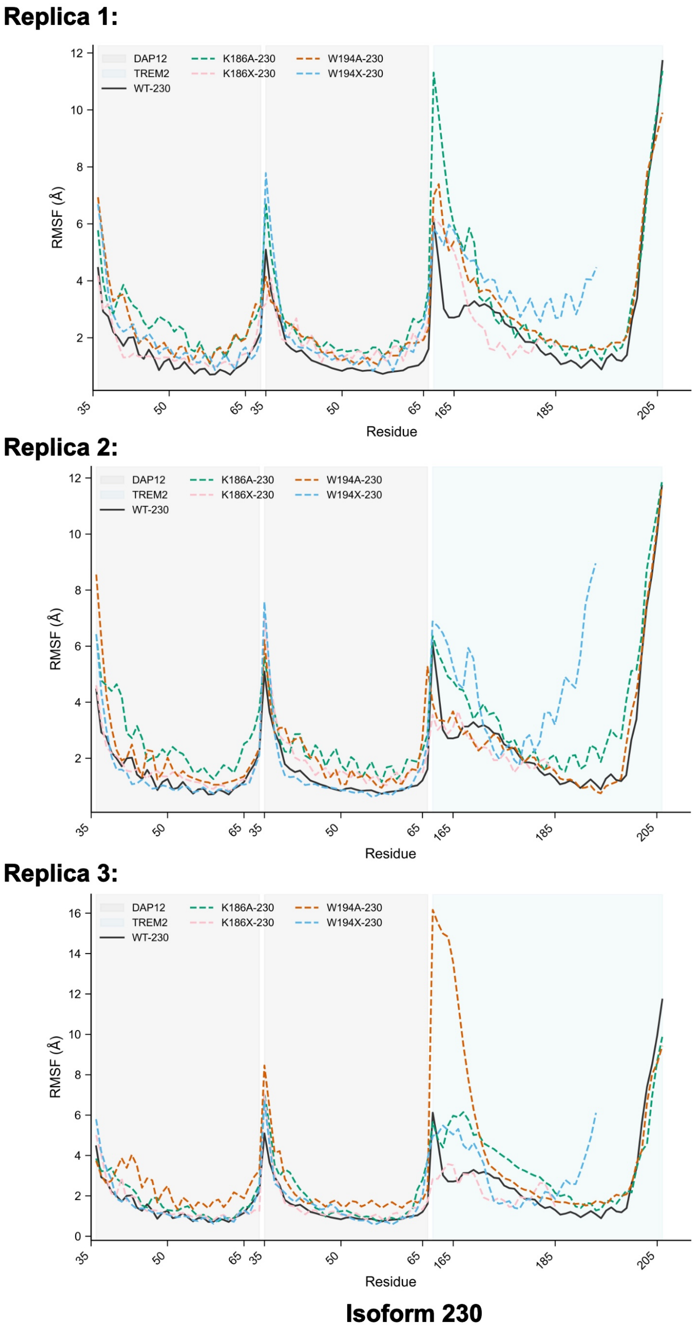

Figure S32: Root mean square fluctuation (RMSF) profiles of the TREM2–DAP12 isoform-230 complexes across three independent replicas. Each panel (Replica 1–3) shows per-residue RMSF values for wild-type (WT-230) and mutants (K186A, K186X, W194A, W194X) after least-squares alignment to the DAP12 transmembrane Cα atoms. Shaded background regions denote DAP12 and TREM2 transmembrane segments. The consistency across replicas confirms the reproducibility of the flexibility patterns discussed in Fig. 6.

Replica 1:

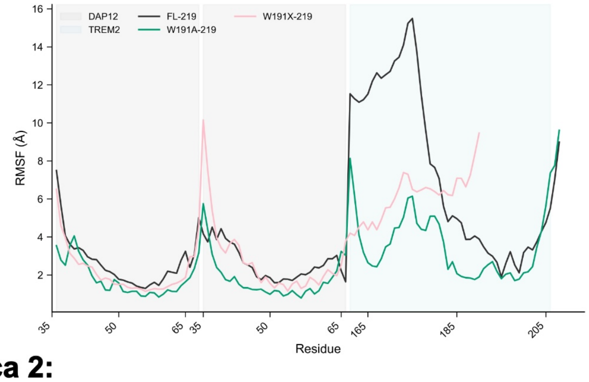

Replica 2:

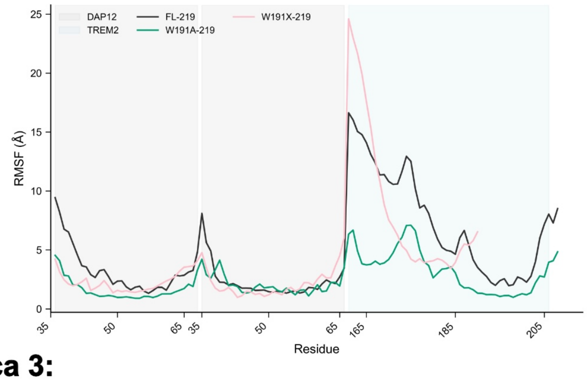

Replica 3:

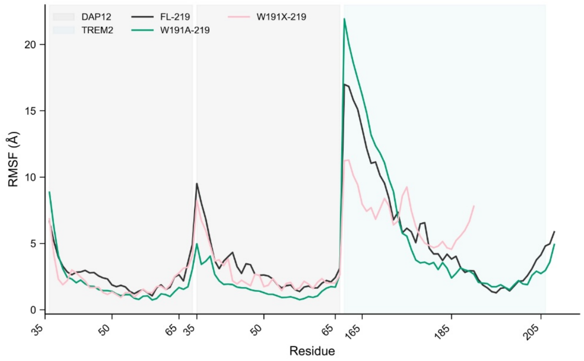

Isoform 219

Figure S33: Root mean square fluctuation (RMSF) profiles of the TREM2–DAP12 isoform-219 complexes across three independent replicas. Each panel (Replica 1–3) shows per-residue RMSF values for wild-type (FL-219) and mutants (W191A, W191X) after least-squares alignment to the DAP12 transmembrane Cα atoms. Shaded background regions denote DAP12 and TREM2 transmembrane segments. The reproducibility across replicas supports the consistency of the flexibility trends presented in Fig. 6.

Table S1: Persistent residue-residue contact frequencies.

	60%	70%	80%	90%
**K186A-230**	I182/V47, F183/T54, L193/A58, W194/V61, W194/Y62, A197/Y62, W198/L66	W194/V61, W194/Y62, A197/Y62, W198/R67	W194/V61, W194/Y62, A197/Y62,	W194/V61, W194/Y62
**K186X-230**	R161/D50, P172/V47, P173/T54, I176/L51, L177/I57, A180/V61, I185/Y62, I185/G65, I185/R66	P173/T54, I176/L51, L177/I57, I185/Y62, I185/G65, I185/L66	P173/T54, I176/L51, L177/I57, I185/Y62, I185/L66	I185/Y62, I185/L66
**W194A-230**	P170/V42, F183/L53, K186/D50, I187/L53, I187/I57, A190/T54, A190/I57, A190/V61, S191/V61, L193/I57, L193/V61, W194/A58, W194/V61, W194/Y62, A197/V61, W198/Y62, W198/G65, W198/R66	K186/D50, I187/I57, A190/T54, A190/I57, A190/V61, A191/V61, L193/I57, L193/V61, W194/A58, W194/V61, A197/V61, W198/Y62, W198/G65, W198/R66	K186/D50, I187/I57, A190/T54, A190/I57, A190/V61, L191/V61, L193/I57, W194/A58, W194/V61, W198/Y62, W198/G65	K186/D50, I187/I57, A190/T54, A194/A58, A194/V61, W198/Y62
**W194X-230**	L177/T54, L178/T54, L178/I57, C181/T54, I182/V61, I185/A58, I185/Y62	L178/T54, L178/I57, I182/V61, I185/A58, I185/Y62	L178/T54, I182/V61, I185/A58, I185/Y62	L178/T54
**FL-219**	W191/T54, G194/T54, L195/I57, L198/A58, L198/V61, L199/V61, W200/R66, Q201/R66, E202/R66	W191/T54, L195/I57, L198/A58, E202/R66	L198/A58, E202/R66	L198/A58
**W191A-219**	K167/D50, L195/I57I, V197/V55, L198/I57, L198/A58, L198/V61, L198/Y62, Q201/Y62, E202/Y62, E202/R66	V197/V55, L198/I57, L198/A58, L198/V61, L198/Y62, Q201/Y62, E202/Y62, E202/R66	L198/A58, L198/V61, Q201/Y62, E202/Y62	L198/A58, E202/Y62
**W191X-219**	None	None	None	None

	20%	30%	40%
**W191X-219**	R161/Y62, R161/R66, R161/L67, V178/L51, P180/D50, P180/L51	R161/Y62, R161/R66, R161/L67	None

Table S2: Sequence of three systems.

**chains**	**sequence**
DAP12 Chain A	*CSTVSPGVLAGIVVGDLVLTVLIALAVYFLGRL*
DAP12 Chain B	*CSTVSPGVLAGIVVGDLVLTVLIALAVYFLGRL*
TREM2-WT-230	*RSLLEGEIPFPPTSILLLLACIFLIKILAASALWAAAWHGQKPGTH*
TREM2-K186A-230	*RSLLEGEIPFPPTSILLLLACIFLIAILAASALWAAAWHGQKPGTH*
TREM2-W194A-230	*RSLLEGEIPFPPTSILLLLACIFLIKILAASALAAAAWHGQKPGTH*
TREM2-W194X-230	*RSLLEGEIPFPPTSILLLLACIFLIKILAASAL*
TREM2-WT-219	*AERHVKEDDGRKSPGEVPPGTSPACILATWPPGLLVLLWQETTLPEH*
TREM2-W191A-219	*AERHVKEDDGRKSPGEVPPGTSPACILATAPPGLLVLLWQETTLPEH*
TREM2-W191X-219	*AERHVKEDDGRKSPGEVPPGTSPACILAT*

Methods

RMSD analysis

The time-dependent root-mean-square deviation (RMSD) was calculated using the `MDAnalysis.analysis.rms.RMSD` module.[1] All trajectories were fitted to the DAP12 transmembrane Cα atoms to remove overall rotation and translation. The RMSD $\rho(t)$ at time t is defined as

$$\rho(t) = \sqrt{\frac{1}{N}\sum_{i=1}^{N} w_i \left\|\mathbf{x}_i(t) - \mathbf{x}_i^{\mathrm{ref}}\right\|^2},$$

where N is the number of atoms, and the normalized weights w_i are given by $w_i = \frac{w_i'}{\langle w' \rangle}$.

Stable plateaus after approximately 50 ns indicate that each system reached conformational equilibrium before production analysis. RMSD were computed for every 10 frames.

Tilt angle calculation

The transmembrane-helix tilt angle θ was calculated as the angle between the helix-axis vector and the bilayer normal (z-axis). Tilt angles were computed each frame as

$$\theta = \cos^{-1}\left(\frac{\mathbf{v}_{\text{helix}} \cdot \mathbf{z}}{|\mathbf{v}_{\text{helix}}|\,|\mathbf{z}|}\right),$$

where $\mathbf{v}_{\text{helix}}$ is the principal-axis vector of the selected helical segment. Consistent average tilts and steady fluctuations confirm stable membrane insertion throughout the production trajectories. For isoform-230, the helix vector was defined using residues A180 and L184 of TREM2; for isoform-219, by A189 and G194 of TREM2 in the wild type and A185 and A189 of TREM2 in the W191A/X mutants. Tilt angles were computed for every 10 frames.

References

(1) Theobald, D. L. Rapid calculation of RMSDs using a quaternion-based characteristic polynomial. *Foundations of Crystallography* **2005**, *61*, 478–480.
